# Targeting mitofusin 1-mediated mitochondrial dynamics to suppress neuroinflammation and pyroptosis after traumatic brain injury

**DOI:** 10.1093/burnst/tkag011

**Published:** 2026-01-28

**Authors:** Tao Liu, Liang Mi, Bo Chen, Meng Nie, Shuo Wang, Bo Wang, Yunhu Yu, Rongcai Jiang

**Affiliations:** Department of Neurosurgery, Xuanwu Hospital, Capital Medical University, 45 Changchun Street, Xicheng District, Beijing 100053, China; Department of Neurosurgery, Tianjin Neurological Institute, State Key Laboratory of Experimental Hematology, Laboratory of Post-Neuroinjury Neurorepair and Regeneration in Central Nervous System Tianjin and Ministry of Education, Tianjin Medical University General Hospital, 154 Anshan Road, Heping District, Tianjin 300052, China; Department of Neurosurgery, Xuanwu Hospital, Capital Medical University, 45 Changchun Street, Xicheng District, Beijing 100053, China; Department of Neurosurgery, Tianjin Neurological Institute, State Key Laboratory of Experimental Hematology, Laboratory of Post-Neuroinjury Neurorepair and Regeneration in Central Nervous System Tianjin and Ministry of Education, Tianjin Medical University General Hospital, 154 Anshan Road, Heping District, Tianjin 300052, China; Department of Neurosurgery, Tianjin Neurological Institute, State Key Laboratory of Experimental Hematology, Laboratory of Post-Neuroinjury Neurorepair and Regeneration in Central Nervous System Tianjin and Ministry of Education, Tianjin Medical University General Hospital, 154 Anshan Road, Heping District, Tianjin 300052, China; Department of Neurosurgery, Tianjin Neurological Institute, State Key Laboratory of Experimental Hematology, Laboratory of Post-Neuroinjury Neurorepair and Regeneration in Central Nervous System Tianjin and Ministry of Education, Tianjin Medical University General Hospital, 154 Anshan Road, Heping District, Tianjin 300052, China; Department of Neurosurgery, The People’s Hospital of Hong Hua Gang District of Zun Yi, No. 185, Wanli Road, Honghuagang District, Zunyi 563000, Guizhou, China; Department of Neurosurgery, Xuanwu Hospital, Capital Medical University, 45 Changchun Street, Xicheng District, Beijing 100053, China; Department of Neurosurgery, Tianjin Neurological Institute, State Key Laboratory of Experimental Hematology, Laboratory of Post-Neuroinjury Neurorepair and Regeneration in Central Nervous System Tianjin and Ministry of Education, Tianjin Medical University General Hospital, 154 Anshan Road, Heping District, Tianjin 300052, China

**Keywords:** Traumatic brain injury, NLRP3 inflammasome, Pyroptosis, Mitochondrial dynamics, Metformin, Mitofusin 1

## Abstract

**Background:**

Traumatic brain injury (TBI) can cause neuroinflammation and neuronal death. The role of mitochondrial dysfunction in regulating inflammasome activation during TBI remains unclear. This study aims to explore mitochondrial regulation of neuroinflammation and pyroptosis after TBI.

**Methods:**

We used a mouse TBI model and *in vitro* scratch-injured HT22 cells and primary neurons to examine changes in mitochondrial dynamics and nucleotide-binding oligomerization domain-like receptors family pyrin domain-containing 3 (NLRP3) inflammasome activation. Metformin treatment, mitofusin 1 (Mfn1) knockdown and regulation of the Mfn1 pathway were applied to evaluate the therapeutic effects and mechanisms.

**Results:**

TBI triggered NLRP3 inflammasome activation and neuronal pyroptosis, along with impaired mitochondrial function and oxidative stress. Metformin reduced inflammasome activation, improved mitochondrial homeostasis, and alleviated neuronal injury. These effects were lost when Mfn1 was silenced, highlighting its essential role. Furthermore, we determined that the AMPK pathway modulates these observed effects.

**Conclusion:**

Metformin protects against TBI-induced neuronal damage by restoring Mfn1-dependent mitochondrial dynamics and suppressing inflammasome activation. Mfn1 is a key mediator linking mitochondrial health to neuroinflammatory responses in TBI.

## Highlights

- Metformin alleviates neuroinflammation and neuronal pyroptosis after TBI.
- TBI disrupts mitochondrial dynamics by suppressing Mfn1 and increasing Drp1 phosphorylation.
- Metformin restores mitochondrial homeostasis through Mfn1-dependent fusion regulation.
- Mfn1 knockdown abolishes the protective effects of metformin on mitochondrial function and inflammasome activation.
- AMPK pathway mediates metformin-induced upregulation of Mfn1 and suppression of NLRP3 inflammasome.

## Background

Traumatic brain injury (TBI) is a leading cause of mortality and long-term neurological disability worldwide [1, 2], and is characterized by both immediate mechanical damage and progressive secondary injury [3, 4]. Beyond the initial trauma, a cascade of secondary events—such as oxidative stress, neuroinflammation, and neuronal cell death—significantly contributes to the deterioration of brain function [3, 5]. Notably, mitochondrial dysfunction and aberrant inflammatory responses have emerged as critical drivers of TBI-induced neuronal loss [6, 7]. Despite ongoing progress in clinical management, current therapeutic approaches remain largely supportive and fail to effectively prevent or reverse the progression of secondary brain injury. These limitations underscore the pressing need for a deeper understanding of TBI pathophysiology and the development of novel intervention strategies targeting the underlying molecular mechanisms.

Mitochondria are essential for maintaining neuronal health, as they provide energy, regulate cellular metabolism, and help control oxidative stress [8]. In healthy cells, mitochondria constantly undergo fusion and fission—a dynamic process that ensures their proper function and distribution [9, 10]. This balance, known as mitochondrial dynamics, is critical for sustaining energy production and responding to cellular stress.

However, following TBI, mitochondrial homeostasis is often disrupted. Excessive mitochondrial fission and impaired mitochondrial fusion lead to fragmented and dysfunctional mitochondria, which results in energy deficits and increased oxidative stress [11]. Damaged mitochondria also release a group of molecules called mitochondrial damage-associated molecular patterns (mtDAMPs), including mitochondrial deoxyribonucleic acid (mtDNA), mitochondrial reactive oxygen species (mtROS), and ATP [12]. These molecules can leak into the cytoplasm or extracellular space and activate multiple inflammatory pathways, such as nucleotide-binding oligomerization domain-like receptors (NLRs), Toll-like receptors (TLRs), and the cGAS–STING pathway [13–16]. Activation of these pathways promotes the release of proinflammatory cytokines and further oxidative damage, resulting in vicious cycle of neuroinflammation and neuronal injury.

Among these inflammatory responses, the NLRP3 inflammasome plays a vital role [17]. It is highly sensitive to mitochondrial-derived danger signals such as mtROS and mtDNA [18]. Once activated, nucleotide-binding oligomerization domain-like receptors family pyrin domain-containing 3 (NLRP3) triggers Caspase-1 activation and the release of  interleukin (IL)-1β and IL-18, leading to pyroptosis, a highly inflammatory form of programmed cell death [19, 20]. While both mitochondrial dysfunction and NLRP3 activation contribute to secondary brain injury after TBI, the precise relationship between them remains unclear.

Recent attention has been given to proteins that regulate mitochondrial dynamics, such as mitofusin 1 (Mfn1), a key mediator of mitochondrial fusion. Mfn1 helps preserve mitochondrial structure and function, and its deficiency has been linked to increased mitochondrial fragmentation, oxidative stress, and neuronal vulnerability [21, 22]. Understanding the role of Mfn1 in TBI may provide important insights into how mitochondrial dysfunction contributes to neuroinflammation and open new avenues for therapeutic intervention.

Metformin, a first-line oral antidiabetic agent, is widely used for the treatment of type 2 diabetes mellitus because of its ability to lower blood glucose levels by reducing hepatic gluconeogenesis and improving insulin sensitivity [23]. In addition to its glucose-lowering effects, clinical and experimental evidence suggests that metformin has a broad range of protective effects in cardiovascular, metabolic, and neurodegenerative diseases [24]. These benefits are largely attributed to its anti-inflammatory and antioxidant properties [25], which appear to be independent of its hypoglycemic action.

Metformin has shown neuroprotective effects in various central nervous system (CNS) injury models. It mitigates inflammation by suppressing proinflammatory cytokine production, inhibiting NF-κB signaling, and reducing NLRP3 inflammasome activation [26, 27]. In addition, metformin improves neurological outcomes by limiting neuronal death and promoting recovery through anti-inflammatory and antiapoptotic mechanisms [28]. Recent studies also highlight its role in regulating mitochondrial function, including enhancing mitochondrial biogenesis, activating AMPK signaling, reducing oxidative stress, and restoring mitochondrial membrane potential (MMP) [18, 29, 30].

Given these effects, metformin has attracted interest as a potential therapeutic agent for TBI, a major form of acute CNS damage. Research has shown that metformin administration after TBI reduces microglial overactivation and neuroinflammation, while improving spatial learning and memory in animal models [31–33]. These findings support its therapeutic potential in TBI. However, most existing studies focus on phenotypic outcomes, with limited exploration of the underlying molecular mechanisms, particularly those related to mitochondrial dynamics and NLRP3 inflammasome-mediated neuronal pyroptosis, which are key to neuronal survival and functional recovery after TBI.

These findings suggest that metformin may protect against secondary neuronal injury by preserving mitochondrial homeostasis and suppressing inflammation. On the basis of these insights, it is plausible to hypothesize that metformin could attenuate mitochondrial dysfunction and NLRP3 inflammasome-mediated pyroptosis in the context of TBI. Therefore, this study aimed to investigate the neuroprotective effect of metformin in a mouse TBI model and to determine whether its beneficial effects on neuronal survival are associated with the inhibition of NLRP3 inflammasome-mediated neuroinflammation and the restoration of mitochondrial dynamics homeostasis. Special attention was given to the role of Mfn1 in mediating the effects of metformin on mitochondrial dynamics and inflammation regulation.

## Methods

### Animals

Male C57BL/6 mice, aged 8 to 10 weeks and weighing between 25 and 30 grams, were obtained from the Laboratory Animal Center of the Academy of Military Medical Sciences (Beijing, China). Prior to any experimental procedures, all the mice were housed under standardized environmental conditions for at least one week to allow for acclimatization. The housing room was maintained at a stable temperature of 20°C–24°C with the relative humidity controlled to between 50% and 60%, and a 12-h light/dark cycle was used. The animals had unrestricted access to standard feed and clean water throughout the study. All procedures involving animals were reviewed and approved by the Animal Ethics Committee of Tianjin Medical University and were performed in accordance with the guidelines outlined in the National Institutes of Health Guide for the Care and Use of Laboratory Animals (IRB2025-DW-58).

### TBI model establishment

A controlled cortical impact (CCI) model of TBI was established using an electromagnetic precision impactor (eCCI-6.3; Custom Design & Fabrication, USA), as previously described [34, 35]. Briefly, mice were anesthetized with 1.5%–3% isoflurane delivered in a gas and positioned on a thermostatic heating pad to maintain the core body temperature at 37°C ± 0.5°C. The head was secured in a stereotaxic frame, and the scalp was incised to expose the skull.

A circular craniotomy ~3.5 mm in diameter was performed over the right parietotemporal cortex (coordinates: 2.5 mm posterior to the bregma and 2.5 mm lateral to the sagittal suture) using a high-speed drill, taking care not to damage the dura mater. CCI was induced using a 2.5 mm diameter flat-tip impactor with the following parameters: an impact velocity of 5 m/s, a dwell time of 200 ms, and a deformation depth of 2.2 mm. Hematoxylin and eosin (H&E) staining was performed to confirm the consistency and severity of the CCI-induced injury across the animals (Figure S1, see online supplementary material). After injury, the scalp was sutured, and the mice were returned to a heating blanket until they fully recovered from anesthesia. Sham-operated animals underwent the same surgical procedures, including craniotomy, but without cortical impact.

### Metformin administration

After the TBI model was established, the sham and TBI + vehicle groups were injected with 0.9% saline vehicle, and the TBI + metf-ormin group was treated with 200 mg/kg metformin (Metformin, Met, dissolved in 0.9% saline; Cat. No. S1950; Selleck Chemicals, Houston, TX, USA) by intraperitoneal (i.p.) injection 1 h immediately following the establishment of the model. The treatment dose of 200 mg/kg was selected on the basis of its well-documented efficacy and widespread application in rodent models [31, 36, 37]. To determine the optimal dosage, we assessed the gasdermin D (GSDMD) protein levels in the ipsilateral cortex of mice treated with metformin at 50, 100, 200, and 400 mg/kg and reported that 200 mg/kg significantly reduced GSDMD expression while exhibiting favorable pharmacokinetic profile (Figure S2, see online supplementary material), supporting our selection of this dose for subsequent experiments. Subsequent administrations of either the vehicle or metformin were performed once daily if the mice were allowed to survive for >1 day.

### Western blot analysis

Western blotting was performed to assess protein expression in brain tissues following TBI [38]. Mice were euthanized under anesthesia, and brain samples were promptly collected and lysed in RIPA buffer containing protease and phosphatase inhibitors (Beyotime, China). The lysates were subsequently centrifuged at 12 000 × g for 15 min at 4°C, after which the supernatants were collected. Protein concentrations were quantified using a BCA Protein Assay Kit (Thermo Fisher Scientific, USA). Equal amounts of protein (5 μg per lane) were loaded onto SDS–polyacrylamide gels, separated by electrophoresis, and transferred onto polyvinylidene difluoride (PVDF) membranes (Millipore, USA). The membranes were blocked with 5% nonfat milk in TBST (Tris-buffered saline with 0.1% Tween-20) for 1 h at room temperature (RT) and then incubated overnight at 4°C with the following primary antibodies: anti-MFNfn1 (1:1000, DF7543, Affinity); anti-phospho-DRP1 (Ser616) (1:1000, 4494 T, Affinity); anti-GSDMD (1:1000, 20 770, Proteintech); anti-IL-18 (1:1000, ab207323, Abcam); anti-IL-1β (1:1000, A19635, ABclonal); anti-ASC (1:1000, F0468, Selleck); anti-Caspase-1 (1:1000, F0461, Selleck); anti-NLRP3 (1:1000, WL02635, Wanleibio); and anti-β-actin (1:5000, TA-09, ZSGB-Bio), which served as the internal controls.

After being washed with TBST, the membranes were incubated with horseradish peroxidase (HRP)-conjugated secondary antibodies (1:5000, ZB-2301, or ZB-2305, ZSGB-Bio) for 1 h at RT. The protein bands were visualized using enhanced chemiluminescence (ECL) substrate (Millipore, USA) and detected with a Bio-Rad imaging system (Bio-Rad, Hercules, CA, USA). Densitometric analysis was performed using ImageJ software (version 1.46r; NIH, USA). The grayscale intensity of each target protein band was normalized to that of the corresponding internal control band (β-actin). The normalized values were then divided by the mean value of the sham group to obtain the relative expression levels.

### Immunofluorescence staining

Mice were humanely euthanized under anesthesia and analgesia as described previously, followed by transcardial perfusion with cold phosphate-buffered saline (PBS, pH 7.4) and subsequently with 4% paraformaldehyde (PFA) to fix the tissues. The brains were carefully removed, postfixed in 4% PFA overnight at 4°C, and then cryoprotected in 30% sucrose solution until they sank. The tissues were embedded in OCT compound (Sakura, Japan) and coronally sectioned at a thickness of 8 μm using a cryostat maintained at −20°C. Sections were mounted on poly-L-lysine-coated glass slides and stored at −80°C until further use. For immunostaining, the brain sections were first rinsed in PBS and permeabilized with 0.1% Triton X-100 (Sigma–Aldrich) for 20 min at RT. Nonspecific binding was blocked by incubation in 3% bovine serum albumin (BSA) for 1 h at RT. The sections were then incubated overnight at 4°C with the following primary antibodies diluted in 1% BSA: anti-NeuN (1:500, 4 047 973, Millipore); anti-GFAP (1:500, 3607, Cell Signaling Technology); anti-Iba-1 (1:500, ab5076, Abcam); anti-GSDMD (1:400, ab219800, Abcam or 1:400, 20 770, Proteintech); anti-NLRP3 (1:300, A5652, ABclonal); anti-ASC (1:300, F0468, Selleck); and anti-phospho-DRP1 (Ser616, 1:300, 4494 T, Affinity).

After primary antibody incubation, the sections were washed with PBS and incubated with species-appropriate Alexa Fluor-conjugated secondary antibodies (1:1000; Invitrogen) for 1 h at RT in the dark. The cell nuclei were counterstained with DAPI (Abcam), and images were captured using an inverted fluorescence microscope (Olympus, Japan). Quantitative analyses of fluorescence intensities and cell counts were performed on randomly selected fields of view using ImageJ software (version 1.46r; NIH, USA).

### HT22 cell culture

HT22 mouse hippocampal neurons were provided by the Cell Center of the Chinese Academy of Sciences (Shanghai, China). The HT22 cells were cultivated in Dulbecco’s modified Eagle’s medium (DMEM, Gibco) supplemented with 10% fetal bovine serum (Gibco) and 1% penicillin–streptomycin mix (No. C0222, Beyotime Biotechnology). The cells were maintained in an incubator at 37°C with 5% CO_2_ and plated for 24 h for adhesion, after which they were subjected to follow-up experiments.

### Primary cortical neuron culture

Primary cortical neurons were isolated from embryonic Day 15.5 (E15.5) mouse embryos [39]. Pregnant mice were sacrificed by cervical dislocation, and the uterine horns were collected under sterile conditions in cold Hank’s balanced salt solution (HBSS). The embryos were removed and decapitated, and the brains were quickly dissected in fresh, cold HBSS. Cerebral cortices were isolated under a stereomicroscope, and the meninges were carefully stripped using fine forceps. The cortices were incubated in 0.25% trypsin–EDTA (Gibco) at 37°C for 15 min. Digestion was stopped by the addition of an equal volume of MEM containing 10% heat-inactivated horse serum (MEM–HS). After two washes with MEM–HS, the tissue was gently triturated with fire-polished Pasteur pipettes to obtain a single-cell suspension. Cells were resuspended in MEM–HS and seeded at a density of 7 × 10^5^ cells per well in poly-L-lysine-coated six-well plates. The cultures were incubated at 37°C with 5% CO₂ for 5–6 h to allow cell attachment. The medium was then replaced with prewarmed N2-supplemented MEM (N2-MEM), and the neurons were maintained under standard conditions for 18–24 h before further use.

### *In vitro* scratch injury model

HT22 mouse hippocampal neuronal cells/primary cortical neurons were cultured in poly-D-lysine-coated 6-well plates with DMEM supplemented with 10% FBS and 1% penicillin–streptomycin. After the neurons covered the entire field of view, mechanical injury was induced by creating a crosshatched scratch pattern using a 100-μL sterile pipette tip, with a spacing of ~3 mm between the scratches. The medium was gently removed to eliminate detached cells and debris and replaced with fresh neurobasal medium. The cells were then incubated under standard conditions for further treatment or analysis. To validate the relevance of this model to TBI, we assessed the expression levels of inflammatory cytokines, including TNF-α, IL-1β, and IL-6—key mediators known to be upregulated—following TBI as part of the neuroinflammatory response [40–42] (Figure S3, see online supplementary material).

### Enzyme-linked immunosorbent assay

To quantify inflammatory cytokines, the levels of TNF-α, IL-1β, and IL-6 in the mouse brain tissue lysates were measured using enzyme-linked immunosorbent assay kits (mlbio, China) following the instructions provided by the manufacturer. The specific catalog numbers used were TNF-α (mI098430), IL-1β (ml106733), and IL-6 (mI098430). Optical densities were recorded at 450 nm using a microplate spectrophotometer. The sample concentrations were determined by comparing the absorbance values to standard curves that were generated from known concentrations.

### Flow cytometry for mitochondrial reactive oxygen species and membrane potential detection

To assess the mitochondrial oxidative stress and membrane potential, MitoSOX™ Red and JC-1 fluorescent probes (Beyotime, China) were used for flow cytometric analyses. mtROS levels were measured using MitoSOX™ Red (Cat# S0061S; Beyotime). Briefly, 1–2 × 10^6^ cells were collected and centrifuged at 600 × g for 5 min at RT. The cell pellet was resuspended in MitoSOX™ Red working solution, which was prepared by diluting MitoSOX™ Red stock solution (5 mm) at 1:1000 in PBS to obtain a final concentration of 5 μm. The cells were incubated at 37°C for 30 min in the dark. Following incubation, cells were washed twice with cold PBS (600 × g, 4°C, 3–4 min per wash), resuspended in PBS, and subjected to flow cytometry. The fluorescence signals were detected using a 405 nm excitation laser and a 610 nm emission filter. The staining concentration and incubation time were optimized on the basis of preliminary experiments. The MMP (ΔΨm) was evaluated using a JC-1 assay kit (Cat# C2003S; Beyotime) [43, 44]. After treatment, 1–2 × 10^6^ cells were harvested and incubated with JC-1 staining solution (as provided in the kit) at 37°C for 20 min in the dark. The cells were then washed twice with JC-1 staining buffer and resuspended in the same buffer for analysis. JC-1 forms red fluorescent aggregates (Ex/Em = 585/590 nm) in healthy mitochondria with high membrane potential, whereas in depolarized mitochondria, it remains in the green fluorescent monomeric form (Ex/Em = 514/529 nm). The red-to-green fluorescence intensity ratio was used to evaluate changes in ΔΨm. Flow cytometry was performed using appropriate dual-channel settings to simultaneously detect both green (FITC channel) and red (PE or PI channel) fluorescence signals.

### Confocal microscopy for mitochondrial visualization and membrane potential analysis

The MMP and morphology were evaluated using MitoTracker Green (Cat# C1048; Beyotime, China) and a JC-1 MMP Assay Kit (Cat# C2003S; Beyotime, China) following the manufacturer’s instructions [45, 46]. Cells were seeded on sterilized glass coverslips and treated according to the experimental design. To assess the MMP (ΔΨm), the cells were subsequently incubated with JC-1 working solution at 37°C for 20 min, washed with JC-1 buffer, and analyzed without fixation. JC-1 exhibits potential-dependent fluorescence: red fluorescence (Ex/Em = 585/590 nm) indicates polarized mitochondria (J-aggregates), whereas green fluorescence (Ex/Em = 514/529 nm) represents depolarized mitochondria (monomers). Fluorescence imaging was performed using an Olympus FV3000 laser scanning confocal microscope. The green and red fluorescence channels were acquired under identical settings, and the images were analyzed using ImageJ software. For mitochondrial staining, the cells were incubated with MitoTracker Green working solution (100 nM in serum-free medium) at 37°C for 30 min in the dark. After being washed twice with PBS, the live cells were immediately imaged to avoid fluorescence loss due to fixation. All staining and imaging procedures were conducted under light-protected conditions.

### Assessment of neurological function

Neurological deficits were evaluated using the modified neurological severity score (mNSS) [42, 47]. Assessments were conducted on postinjury Days 1, 3, 5, 7, and 14 to monitor functional outcomes after TBI. The mNSS is a composite scoring system that includes motor function, sensory response, reflexes, and balance ability. Each abnormal finding or absence of a tested reflex is awarded one point, with total scores ranging from 0 to 14. A score of 0 indicates normal neurological function, whereas higher scores reflect increasing severity: 1–4 (mild), 5–9 (moderate), and 10–14 (severe impairment). All behavioral assessments were independently performed by two experimenters who were blinded to the treatment groups to ensure objectivity.

### Rotarod test

Motor coordination and balance were assessed using an accelerating rotarod apparatus (Ugo Basile, Italy). Tests were conducted on Days 1, 3, 5, 7, and 14 following CCI or sham surgery. For each test session, the mice were placed on a rotating rod that gradually accelerated from 4 to 40 revolutions per minute (rpm) over a maximum duration of 5 min. Each mouse underwent three consecutive trials per time point, with an intertrial interval of 5 min to minimize fatigue. The latency to fall from the rod was automatically recorded for each trial, and the average time of the three trials was calculated for statistical analysis. This test was performed in a quiet environment, and all the assessments were carried out by experimenters who were blinded to the group assignments.

### Morris water maze test

Spatial learning and memory abilities were evaluated using the Morris water maze (MWM) test from Day 15 to Day 21 after TBI. The experimental setup consisted of a circular metal pool (diameter: 120 cm; depth: 50 cm) that was filled with water and rendered opaque using nontoxic white paint. The pool was divided into four equal quadrants, and a hidden circular platform (diameter: 10 cm) was placed 1 cm below the water surface in the center of one quadrant. All tests were conducted in a dimly lit, sound-controlled room to minimize external disturbances. The behavioral protocol was divided into two phases: a 6-day acquisition (training) phase (Days 15–20 postinjury), followed by a probe test on Day 21. During the training phase, each mouse underwent four trials per day, starting from different quadrants in a randomized order. The mice were allowed up to 90 s to locate and climb onto the hidden platform. If a mouse failed to find the platform within the allotted time, it was gently guided to the platform and allowed to remain there for 15 s. On Day 21, the platform was removed to conduct the probe test. Each mouse was placed into the pool from a fixed starting position located diagonally opposite the original platform location and allowed to swim freely for 90 s. A video tracking system (EthoVision XT 13, Noldus Information Technology, Netherlands) was used to record and analyze the swimming trajectories, escape latency during training, time spent in the target quadrant, frequency of platform site crossings, and swimming speed as indicators of motor function. All assessments were performed by investigators who were blinded to group allocation to minimize bias.

### Open field test

To evaluate locomotor activity and anxiety-like behavior, the mice were individually placed in the center of a square open field apparatus (40 × 40 cm) and allowed to explore freely for a duration of 10 min. The apparatus was surrounded by opaque walls (40 cm in height) to prevent escape. Animal movements were recorded using an automated video-tracking system. The total distance traveled and the amount of time spent in the central zone (defined as the central 20 × 20 cm area) versus the peripheral zone were quantified. Increased time spent in the center is typically interpreted as a reduction in anxiety-like behavior.

### Tail suspension test

Depressive-like behavior was assessed using the tail suspension test. Each mouse was suspended by the tail using adhesive tape that was affixed ~1 cm from the tip and hung at a height of 50 cm above the floor for a total of 6 min. Sessions were recorded, and the duration of immobility, defined as the absence of active escape-oriented movements, was measured. Increased immobility time is considered to indicate behavioral despair.

### Novel object recognition test

To assess recognition memory, the novel object recognition (NOR) test was performed following a modified protocol based on Ennaceur and Delacour. The mice were first habituated to an opaque arena (50 × 50 cm) for 10 min. After a 5-min rest in the home cage, two identical objects were placed in the arena, and the mice were allowed to explore them for 5 min. Following another 5-min interval, one familiar object was replaced with a novel object differing in shape, color, and material. The mice were then reintroduced for a 5-min test session. All the objects and the arena were cleaned with 70% ethanol between trials. Thermal imaging was used to monitor mouse behavior during the test phase. The discrimination index (DI) was calculated to evaluate the recognition memory performance in TBI mice.

### Small interfering ribonucleic acid transfection

Mice were stereotactically injected with 2 μl of Mfn1 or negative control small interfering ribonucleic acid ( (siRNA) (RiboBio, China) into the right cortex of the brain (coordinates: 0.2 mm posterior to the bregma, 2.0 mm lateral to the midline, and 1.0 mm ventral to the skull surface) 7 days before CCI. siRNAs with the following sequences were used to silence Mfn1 expression: si-Mfn1 #1: GCACACTATCAGAGCTAAA; si-Mfn1 #2: CAAGAAATGGCCACTACTT; and si-Mfn1 #3: ACTCAAAGCTCTTAAGAAA. To confirm the knockdown efficiency of Mfn1 siRNA, western blot analysis was performed on cortical tissue, revealing significant reductions in Mfn1 protein levels in the si-Mfn1 group compared with those in the vehicle group (Figure S4, see online supplementary material).

### Real time polymerase chain reaction

Total RNA was isolated using TRIzol reagent (Invitrogen, Thermo Fisher Scientific, USA), and 2 μg of RNA was reverse transcribed into cDNA with a PrimeScript RT Reagent Kit (Takara Bio, Tokyo, Japan) following the manufacturer’s protocol. Quantitative polymerase chain reaction (qPCR) was carried out on a Bio-Rad Real-Time PCR Detection System (Hercules, CA, USA) using SYBR Green PCR Master Mix (Applied Biosystems, Waltham, MA, USA). GAPDH served as the reference gene. Gene expression levels were calculated using the 2^−ΔΔCt method.

The primers used for Mfn1 amplification were as follows: forward 5′-GTCCAGGTGCATAATGGCAGA-3′ and reverse 5′-GCCGAAGA-TTGCAGTGATGG-3′. The primers for GAPDH were as follows: forward 5′-GCCAAGGCTGTGGGCAAGGT-3′ and reverse 5′-TCTCCAGGC-GGCACGCAGA-3′. RNA extraction and qPCR analysis were performed by two investigators in a blinded manner.

### Transmission electron microscopy

Primary neurons were first fixed in 2.5% glutaraldehyde at 4°C overnight and then postfixed in 1% osmium tetroxide for 2 h. Following fixation, the samples were dehydrated through a graded ethanol series and embedded in resin. Ultrathin sections (~50–70 nm) were prepared using an ultramicrotome and stained with uranyl acetate and lead citrate. The sections were examined with a transmission electron microscope (HT7700; Hitachi, Tokyo, Japan), and images were acquired using Digital Micrograph software (Gatan Inc., CA, USA).

### Statistical analyses

All the data are presented as the means ± standard deviations (SDs). Statistical analyses were performed using GraphPad Prism 8.1.2 (GraphPad Software, San Diego, CA, USA). For western blot quantification, the signal intensities were normalized to that of the sham or control group. Data normality was assessed using the Shapiro–Wilk test, and homogeneity of variance was evaluated using Levene’s test. For comparisons involving more than two groups with a normal distribution and homogeneous variance, one-way analysis of variance (ANOVA) followed by Tukey’s post hoc test was used. For time-course experiments (e.g. MWM training), repeated-measures ANOVA followed by Bonferroni post hoc correction was applied. For comparisons between two groups, an unpaired Student’s *t* test was used when the variances were equal, and Welch’s *t* test was used when the variances were unequal. Nonparametric data (e.g. mNSS scores and rotarod performance) were analyzed using the Kruskal–Wallis test followed by Dunn’s multiple comparisons test. A *P*-value < 0.05 was considered to indicate statistical significance.

## Results

### Traumatic brain injury induces NOD-, LRR- and pyrin domain-containing protein 3 inflammasome activation and neuronal pyroptosis

**Figure 1:**
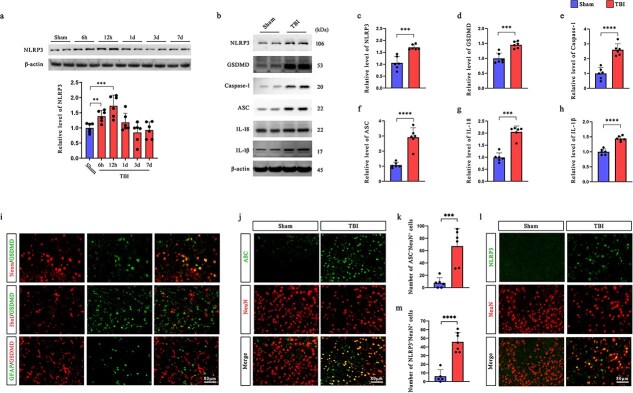
TBI induces NLRP3 inflammasome activation and neuronal pyroptosis. (**a**) Protein levels of NLRP3 in the ipsilateral cortex of mice in the sham group and TBI group at 6 h, 12 h, 1 day, 3 days, and 7 days (*n* = 6). (**b–h**) Protein levels of NLRP3 inflammasome biomarkers (e.g. NLRP3, Caspase-1, ASC, IL-18, and IL-1β) and GSDMD in the ipsilateral cerebral cortex of mice at 12 h after TBI were assessed by western blotting (*n* = 6). (**i**) Colocalization of GSDMD with neurons stained with NeuN (red), microglia stained with Iba-1 (red), and astrocytes stained with GFAP (green). Scale bar: 80 μm. (**j, k**) Representative immunofluorescence images of NeuN and ASC and quantification of the number of ASC^+^NeuN^+^ cells (*n* = 6). Scale bar: 80 μm. (**l, m**) Representative immunofluorescence images of NeuN and NLRP3 and quantification of the number of NLRP3^+^NeuN^+^ cells in the pericontusional cortex of mice at 12 h after TBI (*n* = 6). Scale bar: 80 μm. ^**^P < 0.01, ^***^P < 0.001, ^****^P < 0.0001. *TBI* traumatic brain injury, *NLRP3* nucleotide-binding oligomerization domain-like receptors family pyrin domain-containing 3, *IL* interleukin, *GSDMD* gasdermin D

To investigate whether TBI triggers NLRP3 inflammasome activation, we first measured NLRP3 protein expression in the ipsilateral cortex at multiple time points postinjury. Western blot analysis revealed significant upregulation of NLRP3 expression at 6 and 12 h, peaking at 12 h post-TBI compared with that in the sham group (Figure 1a).

We next assessed the expression of inflammasome-related proteins at 12 h after TBI. Compared with those in the sham control group, the levels of NLRP3, Caspase-1, ASC, IL-18, IL-1β, and GSDMD markedly increased in the TBI group (Figure 1b–h), suggesting robust inflammasome activation and pyroptotic signaling. Consistent trends were also observed in primary neurons, where we validated similar upregulation patterns (Figure S5, see online supplementary material). Immunofluorescence staining further revealed that GSDMD predominantly colocalized with NeuN^+^ neurons rather than microglia (Iba-1^+^ cells) or astrocytes (GFAP^+^ cells), indicating that pyroptosis primarily occurred in neurons 12 h after TBI (Figure 1i).

Moreover, TBI significantly increased the number of ASC^+^ (Figure 1j, k) and NLRP3^+^ (Figure 1l, m) neurons in the pericontusional cortex, providing additional evidence of inflammasome assembly within neurons postinjury. These findings collectively demonstrate that TBI induces NLRP3 inflammasome activation in neurons, as well as pyroptotic cell death and neuroinflammation.

### TBI induces mitochondrial dynamics imbalance and neuronal mitochondrial dysfunction *in vivo* and *in vitro*

**Figure 2:**
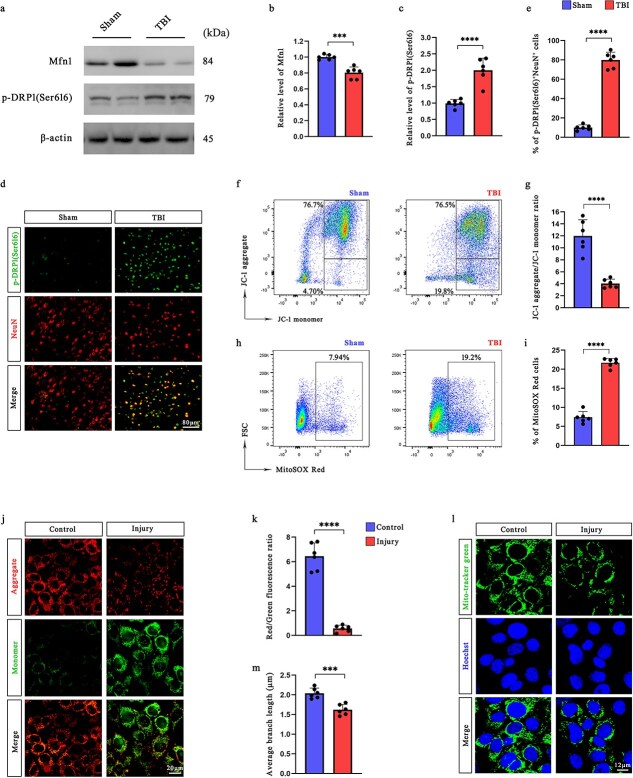
TBI induces mitochondrial dynamics imbalance and neuronal mitochondrial dysfunction in mice and HT22 cells. (**a–c**) Western blotting analysis was used to assess the expression of Mfn1 and p-DRP1 (Ser616) in the ipsilateral cerebral cortex of mice at 12 h after TBI. (**d, e**) Coimmunofluorescence staining for NeuN and p-DRP1 (Ser616) around the lesion and the percentage of p-DRP1 (Ser616)^+^ neurons in the pericontusional cortex of mice at 12 h after TBI (*n* = 6). Scale bar: 80 μm. (**fg**) Cells from the injured hemisphere of TBI mice were stained with JC-1 fluorescent probes, and changes in the MMP were analyzed by flow cytometry (*n* = 6). (**hi**) mtROS levels in the ipsilateral hemisphere of TBI mice were determined using flow cytometry (*n* = 6). (**jk**) Changes in the MMP of HT22 cells were analyzed by JC-1 staining via confocal laser-scanning microscopy (*n* = 6). Scale bar: 20 μm. (**lm**) Mitochondria in HT22 cells were labeled with MitoTracker green, and mitochondrial morphology was analyzed using confocal laser-scanning microscopy. Scale bar: 12 μm. ^***^P < .0.001, ^****^P < 0.0001. *MMP* mitochondrial membrane potential, *TBI* traumatic brain injury, *Mfn1* mitofusin 1

To investigate the impact of TBI on mitochondrial dynamics, we measured the expression of the key regulators of mitochondrial fusion and fission in the ipsilateral cortex at 12 h post-injury. Western blot analysis revealed a significant downregulation of Mfn1, a mitochondrial fusion protein and a marked upregulation of phosphorylated dynamin-related protein 1 at serine 616 [p-DRP1(Ser616)], which is associated with mitochondrial fission (Figure 2a–c). Similar trends were validated in primary neurons (Figure S5, see online supplementary material). These alterations indicate a shift toward mitochondrial fragmentation following TBI.

Immunofluorescence analysis further demonstrated increased p-DRP1 (Ser616) expression in NeuN-positive neurons within the pericontusional cortex of TBI mice. Quantification revealed a significantly greater proportion of p-DRP1(Ser616)-positive neurons than in the sham group (Figure 2d, e), supporting the notion of enhanced mitochondrial fission in neurons after injury. Immunofluorescence colocalization analysis revealed that Mfn1 was primarily localized in neurons 12 h after TBI, as evidenced by its strong colocalization with the neuronal marker NeuN (Figure S6, see online supplementary material), whereas its colocalization with microglial and astrocytic markers was significantly weaker, supporting the neuronal specificity of Mfn1 expression.

To assess mitochondrial function, we evaluated the MMP using JC-1 staining. Flow cytometric analysis demonstrated a significant reduction in the JC-1 aggregate-to-monomer fluorescence ratio in the injured hemisphere, indicating MMP depolarization (Figure 2f, g and Figure S7). In parallel, the mtROS levels were markedly elevated in TBI mice, as shown by increased MitoSOX Red fluorescence intensities (Figure 2h, i and Figure S8a and S9).

Consistent with the *in vivo* findings, HT22 hippocampal neurons that were subjected to injury exhibited similar mitochondrial dysfunction. Confocal microscopy following JC-1 staining revealed a substantial decrease in the red-to-green fluorescence ratio, confirming the loss of the MMP (Figure 2j, k). This phenomenon was further validated in primary neurons that were isolated from mice (Figure S10, see online supplementary material). In addition, MitoTracker Green staining revealed pronounced mitochondrial fragmentation in injured cells, reflecting enhanced mitochondrial fission (Figure 2l, m); these findings were corroborated in primary neurons (Figure S11, see online supplementary material). Moreover, transmission electron microscopy (TEM) of primary neurons revealed marked structural abnormalities, including disrupted and sparse cristae, partial cristae fusion or loss, swollen mitochondrial matrix, and even inner membrane disintegration, in the TBI group compared with the control group (Figure S12, see online supplementary material).

Collectively, these results demonstrate that TBI induces a pronounced imbalance in mitochondrial dynamics, characterized by suppressed fusion and increased fission, leading to mitochondrial dysfunction both *in vivo* and *in vitro* models.

### Administration of metformin suppresses NOD-, LRR- and pyrin domain-containing protein 3 inflammasome activation and neuronal pyroptosis in traumatic brain injury mice

**Figure 3:**
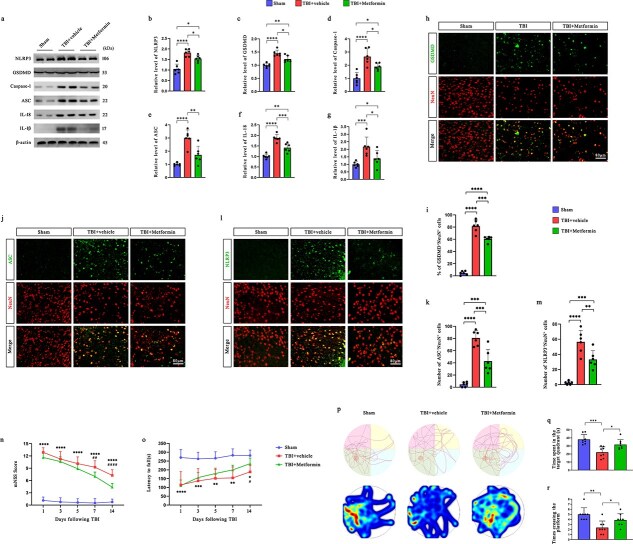
Administration of metformin suppresses NLRP3 inflammasome activation and neuronal pyroptosis in TBI mice. (**a–g**) Protein levels of NLRP3 inflammasome biomarkers (e.g. NLRP3, Caspase-1, ASC, IL-18, and IL-1β) and GSDMD in the ipsilateral cerebral cortex of mice at 12 h after TBI were assessed by western blotting (*n* = 6). (**h–m**) Representative immunofluorescence images of NeuN, GSDMD, ASC and NLRP3 and the percentage of GSDMD^+^NeuN^+^ cells and quantification of the number of ASC^+^ and NLRP3^+^ neurons in the pericontusional cortex of mice at 12 h after TBI (*n* = 6). Scale bar: 80 μm. (**no**) mNSS scores and latency to fall in the rotarod test of mice during a 14-day follow-up period after TBI (*n* = 8). (**p**) Representative swimming traces during the probe trial of the MWM test (last 90 s). (**q, r**) Latency to explore the platform and the number of platform crossings of the TBI mice (*n* = 8). ^*^P < 0.05, ^**^P < 0.01, ^***^P < 0.001, ^****^P < 0.0001. ^#^*P* < 0.05, ^##^*P* < 0.001, ^####^*P* < 0.0001 for the TBI + metformin group compared with the TBI + vehicle group. *TBI* traumatic brain injury, *NLRP3* nucleotide-binding oligomerization domain-like receptors family pyrin domain-containing 3, *IL* interleukin, *GSDMD* gasdermin D

To determine whether metformin modulates NLRP3 inflammasome activity and pyroptotic signaling after TBI, we measured the expression of inflammasome-related proteins in the ipsilateral cortex at 12 h after injury. Western blot analysis revealed that compared with the sham control treatment, the TBI treatment markedly increased the levels of NLRP3, Caspase-1, ASC, IL-1β, IL-18, and GSDMD. Notably, metformin treatment significantly attenuated the upregulation of these proteins (Figure 3a–g), suggesting that metformin suppressed inflammasome activation and pyroptosis. Similar trends were validated in primary neurons (Figure S5, see online supplementary material).

Immunofluorescence further confirmed these findings. Costaining for NeuN and GSDMD revealed a substantial increase in the number of GSDMD^+^ neurons in the pericontusional cortex of TBI mice, which was significantly reduced following metformin administration (Figure 3h, i). Similarly, the increased numbers of ASC^+^ and NLRP3^+^ neurons observed after TBI were also significantly decreased in the TBI + metformin group (Figure 3j–m).

To assess functional outcomes, we conducted behavioral evaluations over a 14-day period. Neurological function, as assessed by the mNSS, was significantly impaired in TBI mice but improved in those treated with metformin (Figure 3n). Motor coordination, evaluated by the results of the rotarod test (Figure 3o), showed a longer latency to fall in the metformin-treated group than in the TBI-vehicle control group.

Cognitive performance was assessed using the MWM test. During the probe trial, TBI mice exhibited disorganized search patterns and impaired spatial memory, as shown by decreased platform crossings and reduced time spent in the target quadrant. These deficits were significantly ameliorated by metformin treatment (Figure 3p–r).

In addition to the MWM test, we conducted complementary behavioral tests to evaluate other cognitive and emotional outcomes after TBI. In the open field test, compared with control mice, TBI mice spent less time in the center area, indicating increased anxiety-like behavior, which was alleviated by metformin (Figure S13a, see online supplementary material). In the NOR test, TBI mice showed impaired recognition memory, as evidenced by a lower DI, whereas metformin significantly improved performance (Figure S13b, see online supplementary material). The tail suspension test (TST) further revealed increased immobility time in TBI mice, reflecting depressive-like behavior, which was also reduced by metformin treatment (Figure S13c, see online supplementary material).

These behavioral improvements were supported by NeuN staining that was performed one month after TBI, which revealed increased neuronal survival in the cortex and dentate gyrus in the metformin group (Figure S13d, see online supplementary material). These findings indicate that metformin has lasting neuroprotective effects after injury.

Collectively, these data demonstrate that metformin mitigates NLRP3 inflammasome activation and neuronal pyroptosis following TBI, ultimately contributing to improved neurobehavioral outcomes.

### Metformin treatment decreases mitochondrial dynamics imbalance and neuronal mitochondrial dysfunction in both *in vivo* and *in vitro* models

**Figure 4:**
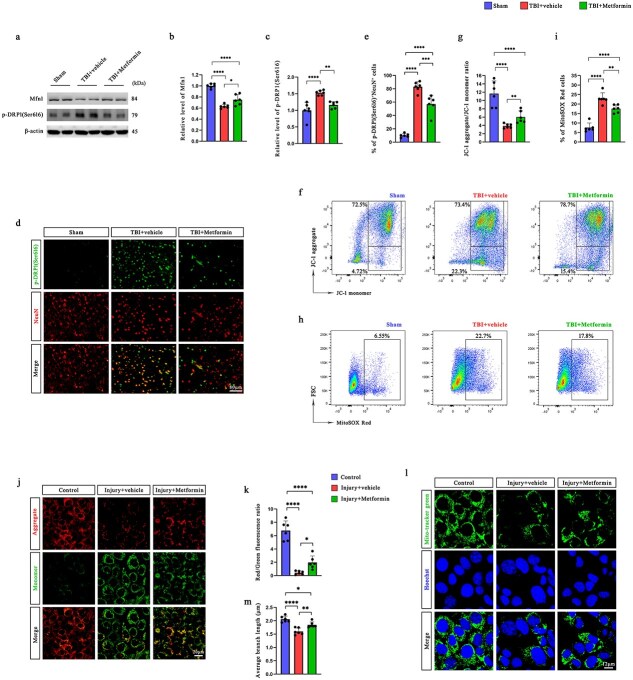
Metformin treatment decreases mitochondrial dynamics imbalance and neuronal mitochondrial dysfunction in TBI mice and scratched HT22 cells. (**a–c**) Western blotting analysis was used to assess the expression of Mfn1 and p-DRP1 (Ser616) in the ipsilateral cerebral cortex of mice at 12 h after TBI. (**de**) Coimmunofluorescence staining for NeuN and p-DRP1 (Ser616) in the pericontusional cortex of mice and the percentage of p-DRP1 (Ser616)^+^ neurons (*n* = 6). Scale bar: 80 μm. (**fg**) Changes in the MMP in the ipsilateral hemisphere of TBI mice were analyzed by flow cytometry (*n* = 6). (**hi**) mtROS levels in the injured hemisphere of TBI mice were determined using flow cytometry (*n* = 6). (**jk**) Changes in the MMP of HT22 cells were analyzed by JC-1 staining via confocal laser-scanning microscopy (*n* = 6). Scale bar: 20 μm. (**lm**) Mitochondria in HT22 cells were visualized through MitoTracker green fluorescence labeling and subsequently examined via confocal laser-scanning microscopy. Scale bar: 12 μm. ^*^P < 0.05, ^**^P < 0.01, ^***^P < 0.001, ^****^P < 0.0001. *MMP* mitochondrial membrane potential, *TBI* traumatic brain injury, *Mfn1* mitofusin 1

To examine the effects of metformin on mitochondrial dynamics following TBI, we first assessed the expression of mitochondrial fusion and fission markers in the injured cortex. Western blot analysis revealed that TBI significantly decreased the level of Mfn1 while increasing the phosphorylation of p-DRP1 (Ser616), indicating enhanced mitochondrial fission. Importantly, metformin treatment partially reversed these changes (Figure 4a–c). We validated the same trend in primary neurons (Figure S5, see online supplementary material). Consistent with these findings, the results of the qPCR analysis revealed a significant reduction in Mfn1 mRNA expression following TBI, which was also partially restored by metformin treatment (Figure S14, see online supplementary material). Immunofluorescence staining further demonstrated elevated p-DRP1 (Ser616) expression in the neurons of the pericontusional cortex after injury, which were significantly reduced by metformin administration (Figure 4d, e), supporting its role in stabilizing mitochondrial dynamics.

To evaluate mitochondrial function, JC-1 staining and flow cytometry were used to assess the MMP. TBI led to a marked decrease in the JC-1 aggregate-to-monomer fluorescence ratio, suggesting that MMP depolarization occurred, whereas metformin treatment significantly increased this ratio (Figure 4f, g and Figure S7, see online supplementary material). In parallel, mitochondrial ROS levels measured by MitoSOX Red staining were significantly elevated in the injured hemisphere, and this increase was attenuated by metformin (Figure 4h, i and Figure S8b, S9, see online supplementary material).

Consistent with the *in vivo* findings, JC-1 staining revealed a pronounced reduction in red fluorescence in HT22 hippocampal neurons after mechanical injury, indicating MMP loss. This effect was partially reversed by metformin, as shown by an increased red/green fluorescence ratio (Figure 4j, k). These results were further validated in primary neurons that were isolated from mice (Figure S5, see online supplementary material). Furthermore, MitoTracker Green staining revealed fragmented mitochondrial morphology in injured cells, which was mitigated by metformin treatment, resulting in a more reticular mitochondrial network (Figure 4l, m). We also validated these findings in primary neurons (Figure S9, see online supplementary material). Furthermore, TEM of primary neurons revealed that compared with the TBI model group, the metformin-treated group exhibited significantly reduced mitochondrial crista disruption. The cristae were more regularly arranged, with less fragmentation and swelling, resembling a near-normal ultrastructural state (Figure S12, see online supplementary material).

Together, these findings demonstrate that metformin restores mitochondrial dynamics homeostasis and alleviates mitochondrial dysfunction in both *in vivo* and *in vitro* TBI models.

### Metformin inhibits NOD-, LRR- and pyrin domain-containing protein 3 inflammasome activation and neuronal pyroptosis via mitofusin 1

**Figure 5:**
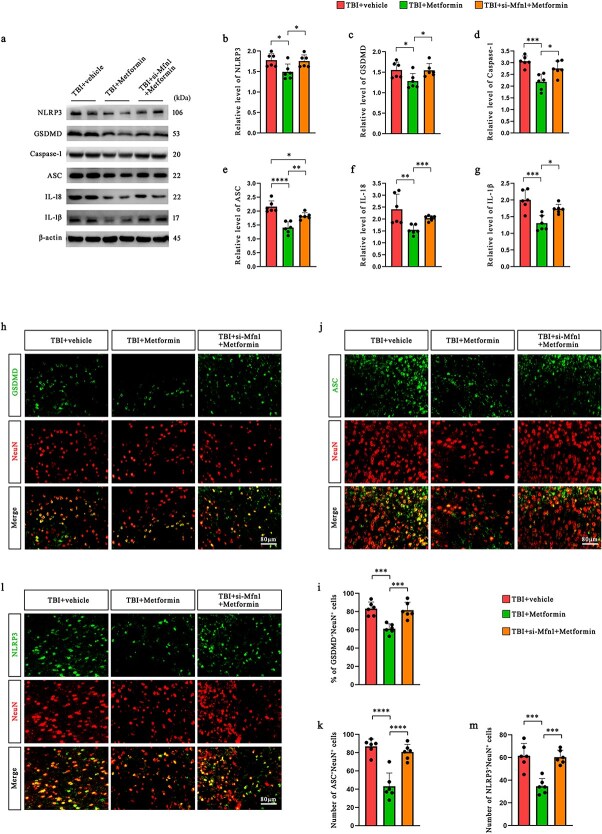
Metformin inhibits NLRP3 inflammasome activation and neuronal pyroptosis via Mfn1. (**a–g**) Protein levels of NLRP3 inflammasome biomarkers (e.g. NLRP3, Caspase-1, ASC, IL-18, and IL-1β) and GSDMD in the pericontusional cortex of mice at 12 h after TBI were assessed by western blotting (*n* = 6). (**h–m**) Representative immunofluorescence images of NeuN, GSDMD, ASC, and NLRP3 and the percentage of GSDMD^+^ neurons and quantification of the numbers of ASC^+^NeuN^+^ cells and NLRP3^+^NeuN^+^ cells in the pericontusional cortex of mice at 12 h after TBI (*n* = 6). Scale bar: 80 μm. ^*^P < 0.05, ^**^P < 0.01, ^***^P < 0.001, ^****^P < 0.0001. *TBI* traumatic brain injury, *NLRP3* nucleotide-binding oligomerization domain-like receptors family pyrin domain-containing 3, *IL* interleukin, *GSDMD* gasdermin D, *Mfn1* mitofusin 1

To investigate whether the protective effects of metformin on inflammasome activation are mediated through its influence on mitochondrial dynamics, we transfected siRNAs to block the production of Mfn1. We assessed key pyroptosis-related proteins in the pericontusional cortex at 12 h post-TBI. Western blot analysis revealed that compared with the TBI + vehicle treatment, metformin treatment significantly reduced the expression of NLRP3, Caspase-1, ASC, IL-18, IL-1β, and GSDMD. However, these inhibitory effects were partially reversed upon Mfn1 knockdown (Figure 5a–g), suggesting an Mfn1-dependent mechanism. Consistent trends were observed and validated in primary neuronal cultures (Figure S15, see online supplementary material).

Immunofluorescence staining further supported these findings. The proportion of GSDMD^+^ neurons was significantly lower in the metformin-treated group than in the TBI alone group, while Mfn1 knockdown partially restored GSDMD expression in neurons (Figure 5h, i). Similarly, the number of ASC^+^NeuN^+^ and NLRP3^+^NeuN^+^ cells markedly decreased following metformin administration, and this effect was attenuated by si-Mfn1 treatment (Figure 5j–m).

These results indicate that metformin inhibits NLRP3 inflammasome activation and neuronal pyroptosis largely through Mfn1-mediated mitochondrial regulation.

### Mitofusin 1 is required for the metformin-mediated preservation of mitochondrial homeostasis and redox balance

**Figure 6:**
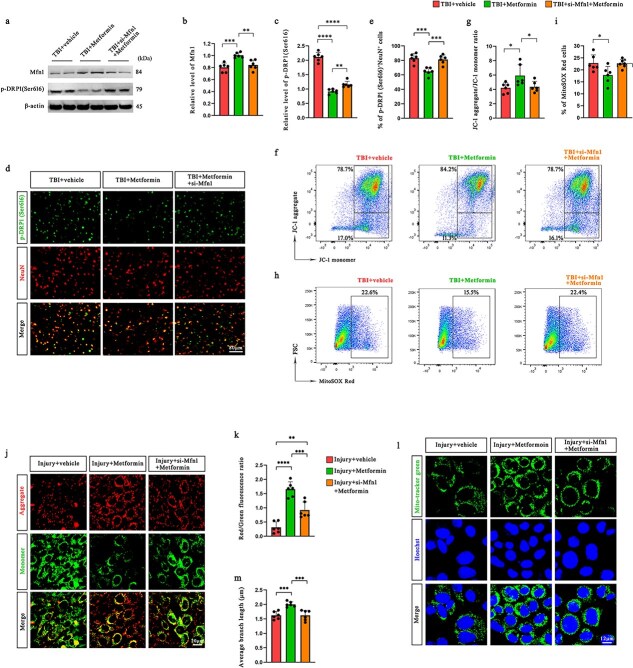
Mfn1 plays a critical role in mediating the effects of metformin on mitochondrial homeostasis maintenance and oxidative stress attenuation in both TBI mice and scratched HT22 cells. (**a–c**) Western blotting analysis was used to assess the expression of Mfn1 and p-DRP1 (Ser616) in the ipsilateral cerebral cortex of mice at 12 h after TBI. (**de**) Coimmunofluorescence staining for NeuN and p-DRP1 (Ser616) in the pericontusional cortex of mice and the percentage of p-DRP1 (Ser616)^+^ neurons (*n* = 6). Scale bar: 80 μm. (**fg**) Changes in the MMP in the ipsilateral hemisphere of TBI mice were analyzed by flow cytometry (*n* = 6). (**hi**) mtROS levels in the injured hemisphere of TBI mice were determined using flow cytometry (*n* = 6). (**jk**) Changes in the MMP of HT22 cells were analyzed by JC-1 staining via confocal laser-scanning microscopy (*n* = 6). Scale bar: 20 μm. (**lm**) Mitochondria in HT22 cells were visualized through MitoTracker green fluorescence labeling and subsequently examined via confocal laser-scanning microscopy. Scale bar: 12 μm. ^*^P < 0.05, ^**^P < 0.01, ^***^P < 0.001, ^****^P < 0.0001. *MMP* mitochondrial membrane potential, *TB*I traumatic brain injury, *Mfn1* mitofusin 1

To clarify the role of Mfn1 in the protective effects of metformin on mitochondria following TBI, we assessed indicators of mitochondrial dynamics and function in both *in vivo* and *in vitro* models. Western blot analysis revealed that metformin treatment significantly upregulated Mfn1 and reduced the phosphorylation of DRP1 at Ser616 in the injured cortex, whereas siRNA-mediated knockdown of Mfn1 abolished these effects (Figure 6a–c). Similar trends were validated in primary neurons (Figure S15, see online supplementary material).

Consistent with these findings, immunofluorescence staining revealed that metformin markedly decreased the number of p-DRP1(Ser616)^+^ neurons in the pericontusional region, an effect that was reversed upon Mfn1 silencing (Figure 6d, e). These results suggest that Mfn1 is essential for the inhibition of mitochondrial fission that is induced by metformin.

Flow cytometric analysis of JC-1 staining indicated that metformin restored the MMP in TBI mice, as evidenced by an increased JC-1 aggregate-to-monomer ratio. This improvement was significantly attenuated in the TBI + metformin + si-Mfn1 group (Figure 6f, g). Similarly, metformin reduced mtROS levels, whereas Mfn1 knockdown abolished this antioxidative effect (Figure 6h, i).

*In vitro*, JC-1 staining of HT22 cells confirmed that metformin restored the red/green fluorescence ratio following injury, indicating improved MMP. However, this restoration was significantly impaired when Mfn1 was silenced (Figure 6j, k). This phenomenon was further validated in primary neurons isolated from mice (Figure S10, see online supplementary material). Moreover, MitoTracker Green staining revealed that metformin alleviated mitochondrial fragmentation in injured HT22 cells, whereas Mfn1 knockdown abolished this morphological rescue (Figure 6l, m); these findings were consistent with those in primary neurons (Figure S11, see online supplementary material). TEM further confirmed these observations at the ultrastructural level (Figure S12, see online supplementary material). Compared with the TBI + metformin group, the TBI + metformin + si-Mfn1 group exhibited sparse and fragmented mitochondrial cristae, a swollen matrix, and pronounced structural abnormalities.

Taken together, these findings demonstrate that Mfn1 is a critical mediator of the protective effects of metformin on mitochondrial dynamics, membrane potential, and oxidative stress in both TBI brain tissue and neuronal cultures.

### Metformin regulates mitofusin 1 and inhibits NOD-, LRR- and pyrin domain-containing protein 3 activation via the AMP-activated protein kinase pathway

**Figure 7:**
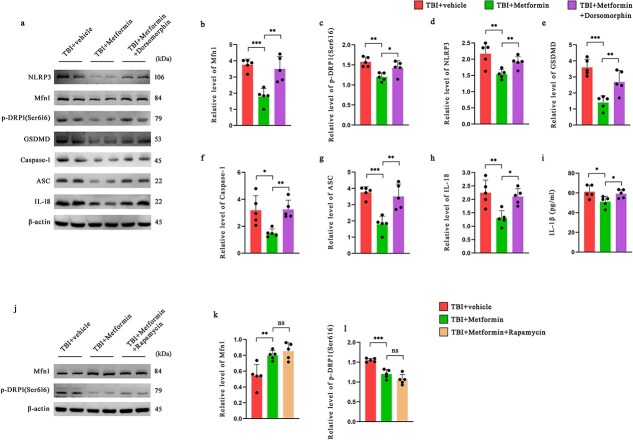
The suppression of NLRP3 inflammasome activation by metformin, achieved through MFN1-mediated regulation of mitochondrial dynamics, is mediated by the AMPK pathway and not by the mTOR pathway. (**a–h**) Western blotting analysis was used to assess mitochondrial dynamics homeostasis and NLRP3 inflammasome activation (*n* = 5). (**i**) ELISA analyses of IL-1β in the ipsilateral cerebral cortex from the indicated groups (*n* = 5). (**j–l**) The protein levels of Mfn1 and p-DRP1 (Ser616) in the ipsilateral cerebral cortex of mice at 12 h after TBI were assessed by western blotting (*n* = 5). ^*^P < 0.05, ^**^P < 0.01, ^***^P < 0.001, ^****^P < 0.0001. *TBI* traumatic brain injury, *NLRP3* nucleotide-binding oligomerization domain-like receptors family pyrin domain-containing 3, *IL* interleukin, *GSDMD* gasdermin D, *Mfn1* mitofusin 1

To explore how metformin regulates Mfn1 expression, we investigated the involvement of the AMPK and mTOR pathways. As shown in Figure 7, metformin treatment increased Mfn1 levels and reduced p-DRP1 (Ser616) levels, suggesting improved mitochondrial dynamics. These effects were blocked by the AMPK inhibitor dorsomorphin (Figure 7a–c) but not by the mTOR inhibitor rapamycin (Figure 7j–l), indicating that metformin acts through the AMPK pathway. In addition, metformin reduced the expression of NLRP3, Caspase-1, GSDMD, ASC, and IL-1β, resulting in the inhibition of inflammasome activation (Figure 7a, e–i). This anti-inflammatory effect was also reversed by dorsomorphin, further supporting the role of AMPK. These results suggest that metformin improves mitochondrial function and suppresses NLRP3 inflammasome activation mainly through AMPK signaling.

## Discussion

In the present study, we comprehensively explored the relationship between mitochondrial dynamics and inflammasome activation in the context of TBI and revealed a previously unrecognized role for Mfn1-mediated mitochondrial fusion as a prerequisite for the neuroprotective efficacy of metformin. Our data establish a mechanistic bridge linking mitochondrial dysfunction, oxidative stress, and NLRP3 inflammasome-driven neuronal pyroptosis in the injured brain. By utilizing both a murine TBI model and an *in vitro* injury model in HT22 hippocampal neurons and primary neurons, we demonstrated that metformin not only ameliorated mitochondrial impairment and restored the MMP but also significantly limited inflammasome activation and pyroptotic neuronal cell death. Crucially, these protective effects were contingent upon the presence of Mfn1, revealing a specific molecular target through which metformin preserves neuronal integrity. Collectively, these findings offer fresh insights into the cellular events underlying secondary brain injury and support the therapeutic potential of metformin as a mitochondria-directed intervention in TBI.

Maintaining mitochondrial homeostasis is vital for neuronal survival, particularly given the high metabolic demand of the brain. After TBI, neurons undergo profound alterations in mitochondrial morphology, typified by excessive fission and reduced fusion [9, 48]. This shift is reflected by decreased levels of Mfn1 and heightened phosphorylation of DRP1 at Ser616 [21, 49]. Such dysregulation leads to mitochondrial fragmentation, MMP dissipation, and excessive generation of mtROS [48, 50, 51]. These mitochondrial disturbances not only compromise bioenergetic function but also promote the release of mtDAMPs, which are key upstream signals for innate immune activation [52]. Our findings are in line with previous reports that mitochondrial dysfunction amplifies neuroinflammation through ROS production and inflammasome priming [53, 54]. Importantly, our findings indicate that Mfn1 functions as a critical regulatory node within this signaling cascade, governing mitochondrial integrity and consequently attenuating downstream inflammasome activation and neuroinflammatory responses. These data suggest that preserving mitochondrial fusion capacity may serve as an effective strategy to disrupt the feed-forward loop of injury-induced inflammation.

The NLRP3 inflammasome serves as a critical sensor of mitochondrial stress, including signals such as mtROS, ATP, and oxidized mtDNA [55, 56]. Upon activation, NLRP3 forms a multiprotein complex with ASC and pro- Caspase-1, culminating in Caspase-1 activation, maturation of IL-1β and IL-18, and membrane permeabilization through GSDMD-mediated pyroptosis [55, 57]. Our results confirm the pronounced activation of the NLRP3 inflammasome following TBI, as evidenced by the upregulated expression of its core components and inflammatory outputs. Notably, these effects were significantly reversed by metformin treatment. However, silencing Mfn1 abolished the suppressive effects of metformin on inflammasome signaling, suggesting that mitochondrial fusion integrity is a prerequisite for inflammasome regulation. This finding is consistent with accumulating evidence suggesting that mitochondrial quality control processes—including mitochondrial fusion and biogenesis—play a pivotal role in restraining aberrant innate immune activation in the context of neurological disorders [58, 59]. Our findings add to this perspective by explicitly demonstrating that Mfn1 serves as a molecular link between mitochondrial structure and inflammatory signaling in neurons.

Although mitochondrial dysfunction and NLRP3 inflammasome activity have both been implicated in TBI pathology [60, 61], the precise crosstalk that occurs between these two processes has remained largely unclear. Prior studies have often examined mitochondrial damage and neuroinflammation in isolation but have not investigated how disturbances in mitochondrial dynamics may directly contribute to inflammasome activation. Our work addresses this gap by demonstrating that Mfn1-mediated mitochondrial fusion plays a pivotal role in modulating both mitochondrial homeostasis and inflammatory outcomes. Furthermore, our data indicate that the neuroprotective effects of metformin extend beyond its broad anti-inflammatory properties and are specifically mediated through the preservation of mitochondrial structural integrity. These findings support the findings of previous studies that reported the anti-inflammatory effects of metformin across various pathological contexts [62, 63], but lacked mechanistic insight into its upstream regulation of mitochondrial dynamics.

In contrast to previous studies that primarily assessed mitochondrial changes in heterogeneous tissue samples, our study employed dual *in vivo* and *in vitro* models, allowing for precise localization and cell-specific analysis. By integrating coimmunofluorescence staining, JC-1-based assays of MMP, mtROS quantification, and siRNA-mediated knockdown of Mfn1, we systematically investigated the neuron-specific contributions of metformin and Mfn1 to mitochondrial homeostasis and inflammatory regulation. This enhanced level of analytical resolution offers deeper mechanistic insight into the cellular pathology of TBI and highlights the translational potential of targeting mitochondrial dynamics as a therapeutic strategy.

To investigate how metformin regulates mitochondrial dynamics, we examined the AMPK and mTOR pathways. Our data revealed that metformin upregulated Mfn1 levels and reduced p-DRP1 (Ser616) levels via AMPK activation but not through mTOR activity. The inhibition of AMPK abolished these effects and reversed the suppression of NLRP3 inflammasome markers, suggesting that AMPK is a key upstream regulator that links metformin to mitochondrial and inflammatory changes. In this study, we explored how molecular and cellular changes are related to behavioral outcomes after TBI. Our results suggest a clear link between molecular mechanisms and functional recovery. At the molecular level, metformin increased the expression of Mfn1 and reduced the phosphorylation of DRP1 (Ser616), helping to restore mitochondrial dynamics. It also decreased the levels of NLRP3, GSDMD, and IL-1β, indicating reduced inflammasome activation and inflammation. These molecular effects supported neuronal function. Improved mitochondrial health and suppressed inflammation contributed to better neuronal survival and synaptic maintenance, as shown by increased NeuN expression in the injured brain regions. These cellular improvements were reflected in behavior. Metformin-treated mice performed better on memory-related tasks (e.g. MWM and NOR) and presented fewer signs of anxiety and depression (e.g. PFT and TST). Together, these findings support a logical pathway through which metformin-mediated molecular regulation improves neuronal function and ultimately leads to better behavioral outcomes.

While metformin may exert neuroprotective effects through multiple pathways, including gut microbiota modulation via the brain–gut axis, our study focused on mitochondrial dynamics because of its early involvement in TBI pathology. Disrupted fusion–fission balance leads to energy failure and neuronal pyroptosis, events that occur upstream or parallel to inflammation. Although the gut-brain axis is important, the role of metformin in regulating mitochondrial dynamics via the AMPK-Mfn1 pathway remains less explored. Our findings address this gap and suggest a direct cellular mechanism underlying the protective effects of metformin. We also acknowledge possible crosstalk between the gut microbiota and mitochondrial function. For example, short-chain fatty acids from gut bacteria can activate AMPK, potentially linking microbial signals to Mfn1-mediated dynamics. Future studies will explore this interaction further. Collectively, these findings not only strengthen the evidence that mitochondrial dysfunction is a key driver of inflammasome-mediated neuronal damage but also identify Mfn1 as a viable therapeutic target. By shifting the therapeutic paradigm from downstream cytokine inhibition to upstream preservation of mitochondrial integrity, our study proposes a novel mechanistic framework for the treatment of TBI and, potentially, other neuroinflammatory disorders.

On the basis of our findings, several future directions could help clarify and expand the therapeutic potential of the AMPK-Mfn1-mitochondrial pathway. First, given the influence of sex hormones on mitochondrial function and inflammation, conducting parallel studies in female mice may reveal sex-specific responses and enhance clinical relevance [64, 65]. Second, we focused primarily on Mfn1; future work should investigate the contributions of other fusion/fission regulators such as OPA1 and Fis1 [66, 67]. Third, combining metformin with other mitochondrial protectants (e.g. coenzyme Q10) or anti-inflammatory agents may provide additive or synergistic effects by targeting multiple aspects of TBI pathology [68]. Finally, validation of this mechanism in other TBI subtypes, such as diffuse axonal injury, or in models of neurodegenerative diseases could support its broader applicability and translational value.

In conclusion, our study revealed that metformin confers neuroprotective effects following TBI by restoring mitochondrial dynamics and suppressing NLRP3 inflammasome activation in an Mfn1-dependent manner. These findings support the therapeutic potential of targeting mitochondrial homeostasis and inflammasome signaling to mitigate neuronal damage after brain injury.

## Conclusions

In conclusion, this study demonstrated that TBI-induced neuronal damage is associated with NLRP3 inflammasome activation, pyroptotic cell death, and mitochondrial dysfunction. Metformin treatment effectively ameliorated these pathological changes by preserving mitochondrial homeostasis and suppressing inflammatory responses. Importantly, we identified Mfn1 as a crucial mediator of the therapeutic benefits of metformin, as evidenced by the loss of protection following Mfn1 knockdown. These findings not only expand our understanding of the mechanistic interplay between mitochondrial dysfunction and neuroinflammation in TBI but also provide new insights into the therapeutic potential of metformin. Our results suggest that targeting mitochondrial dynamics through metformin administration may represent a promising strategy for TBI treatment.

## Supplementary Material

tkag011_Supplemental_Files

## Data Availability

All the data supporting this study are included in the manuscript and supplementary materials or are available upon reasonable request.
