# Supplementary material for: Targeting mitofusin 1-mediated mitochondrial dynamics to suppress neuroinflammation and pyroptosis after traumatic brain injury
Source: Burns Trauma. 2026 Jan 28;14:tkag011. doi: 10.1093/burnst/tkag011 (PMC13152094; doi:10.1093/burnst/tkag011)
Supplement: tkag011_Supplemental_Files [file tkag011_supplemental_files.zip › Supplementary materials.docx]

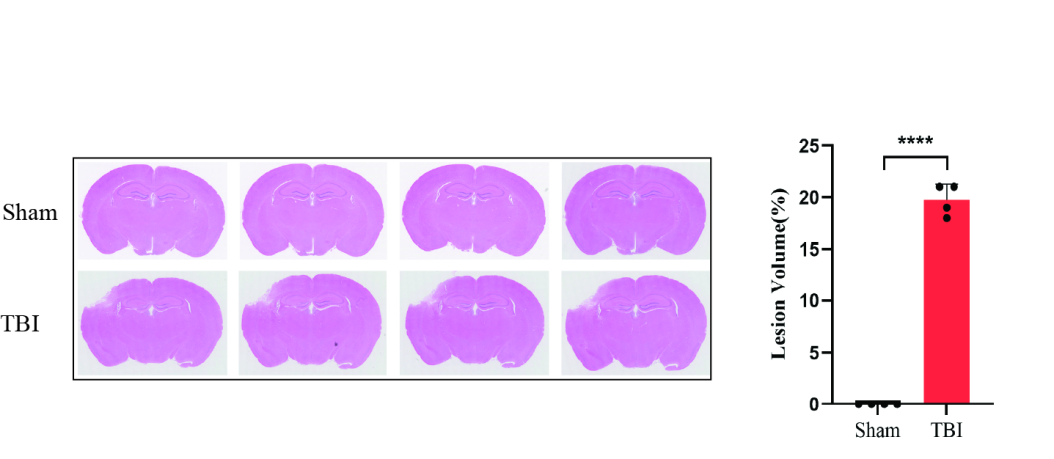


Figure S1. HE-stained coronal sections fromfrom mice in the sham group and 12 hours after CCI impact. The bar chart shows severe tissue defects in the cerebral cortex of mice 12 hours after CCI, and the percentage of brain defect area varies little among different sham group mice, demonstrating the stability of the CCI modeling (n=4). *****p* < 0.0001


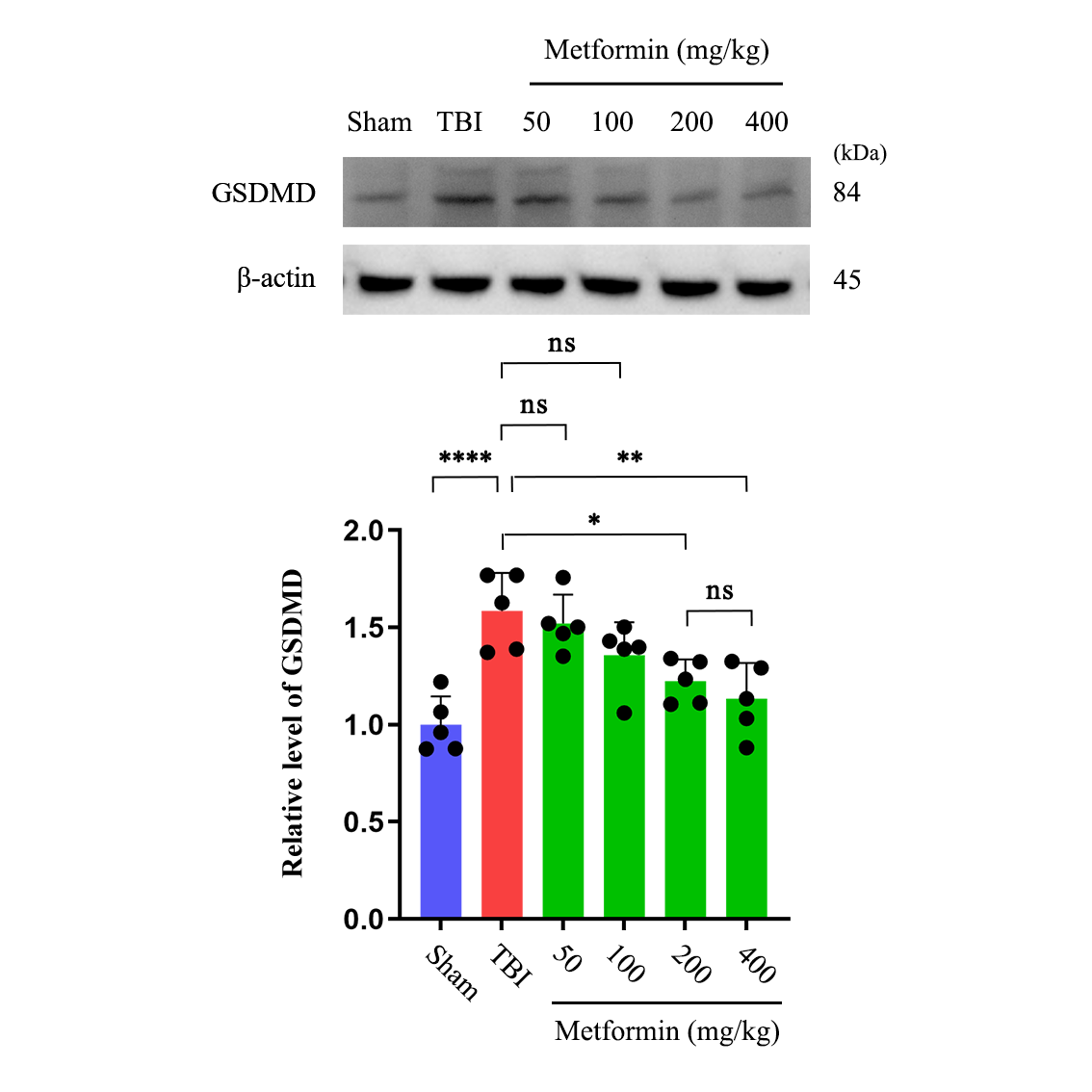


Figure S2. Protein levels of GSDMD in the ipsilateral cortex of mice from sham group and TBI group administered metformin at doses of 50, 100, 200 and 400 mg/kg (n=5). **p* < 0.05, ***p* < 0.01, *****p* < 0.0001


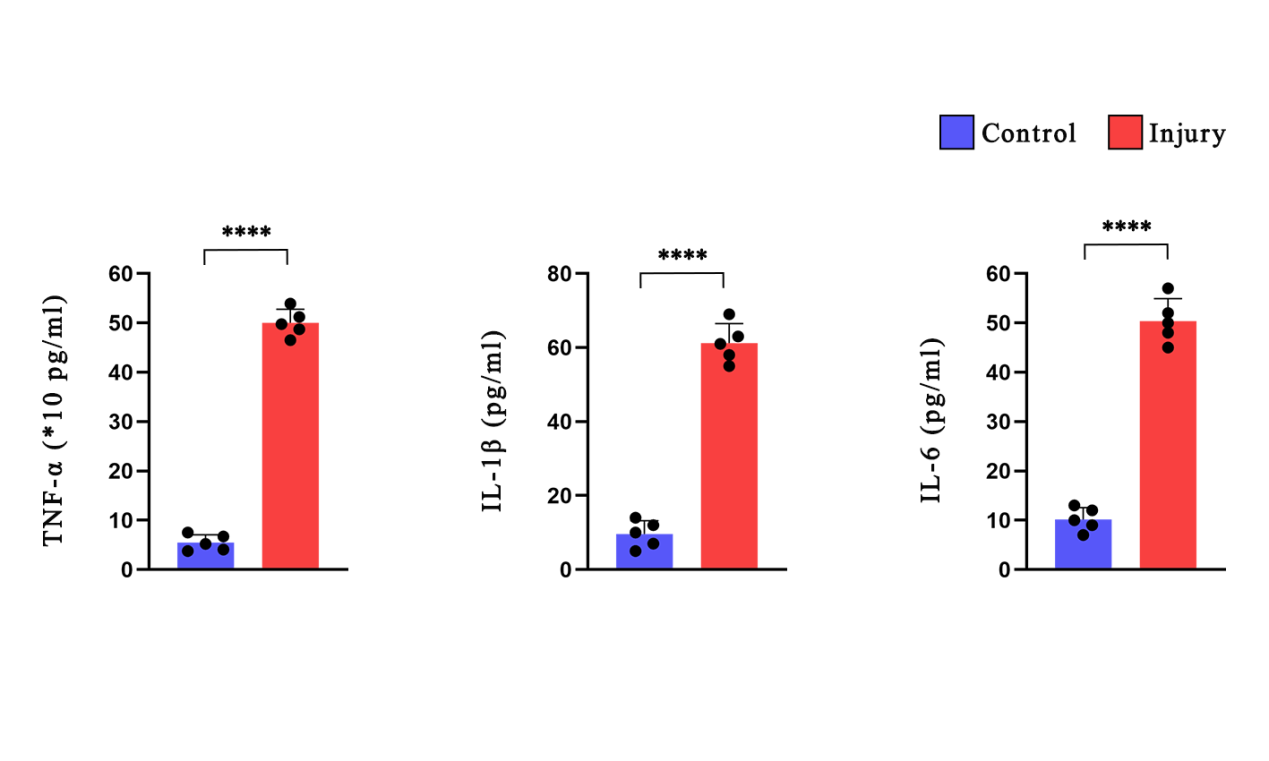


Figure S3. Elisa analyses of pro-inflammatory cytokines (TNF-α, IL-1β and IL-6) in the primary neurons from indicated groups (n=5). *****p* < 0.0001.


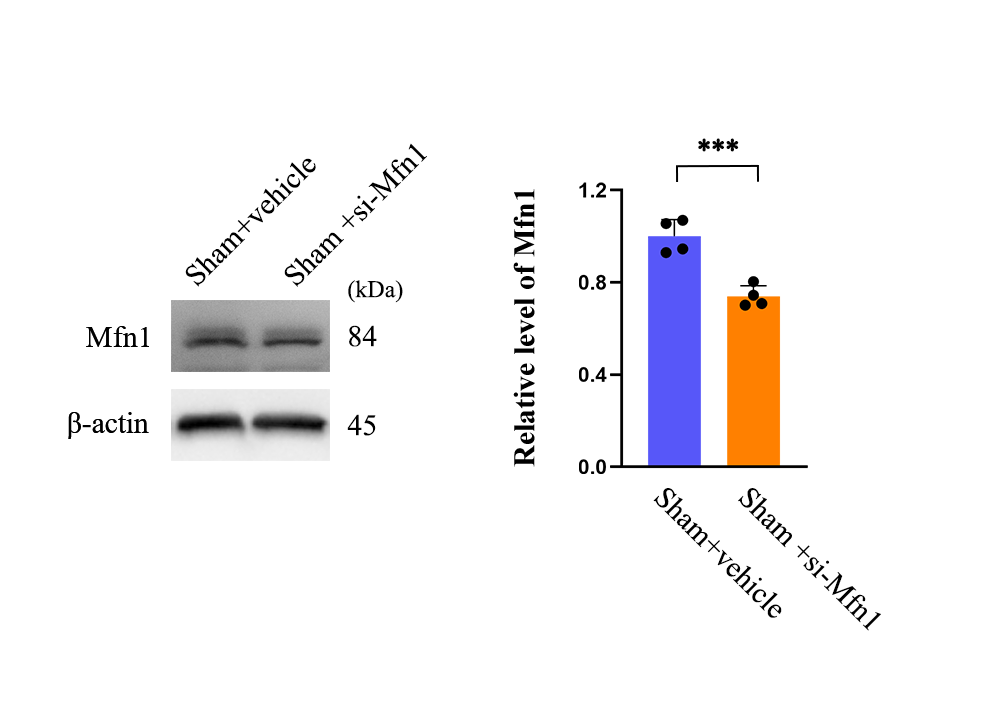


Fig S4. Protein levels of Mfn1 in the cortex of mice from indicated groups (n=4). ****p* < 0.001


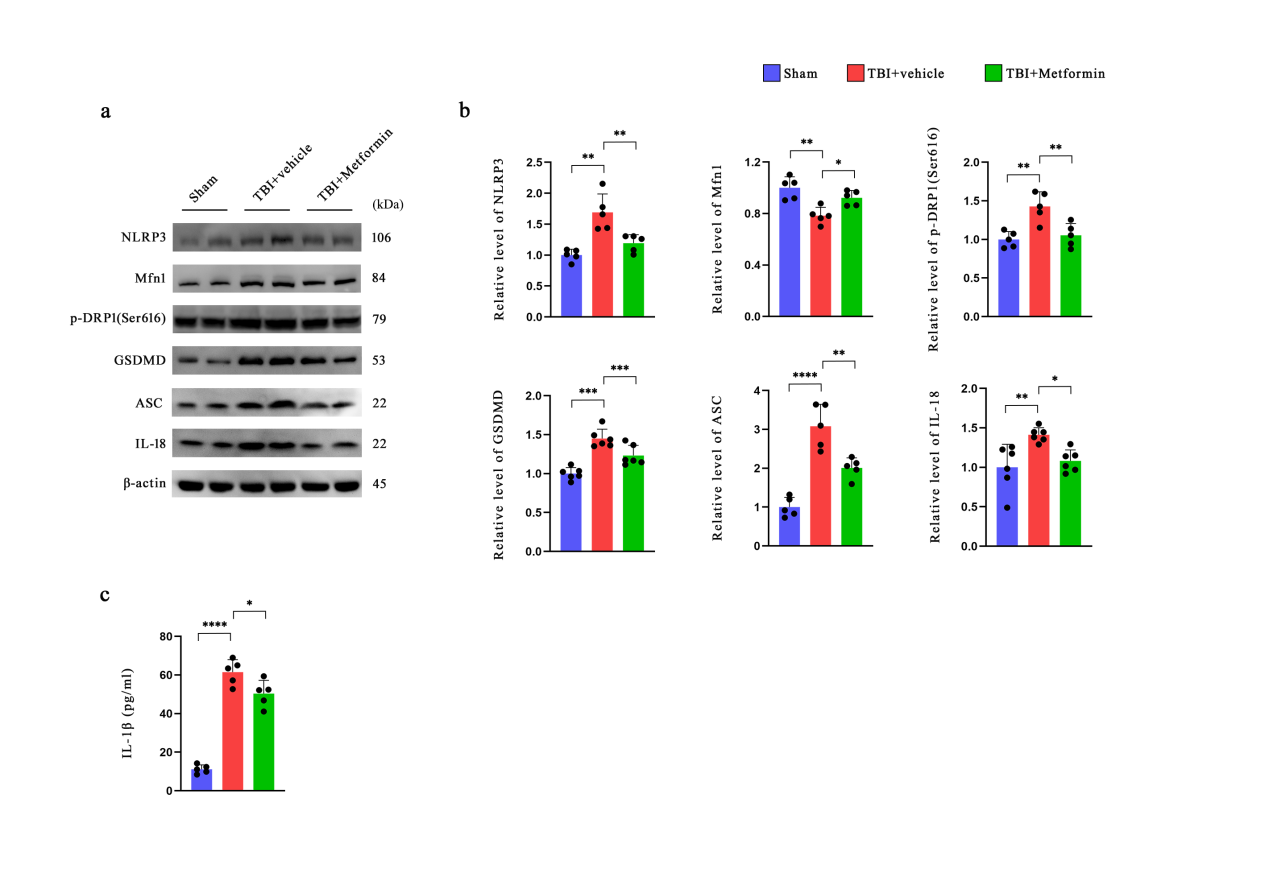


Figure S5. (**a, b**) Protein levels of NLRP3 inflammasome biomarkers (NLRP3, ASC, IL-18), GSDMD and mitochondrial dynamics biomarkers (Mfn1, p-Drp1 (Ser616)) in the primary neurons after scratch assay (n=5). (**c**) Elisa analyses of IL-1β in the primary neurons after scratch assay from indicated groups (n=5). **p* < 0.05, ***p* < 0.01, ****p* < 0.001, *****p* < 0.0001


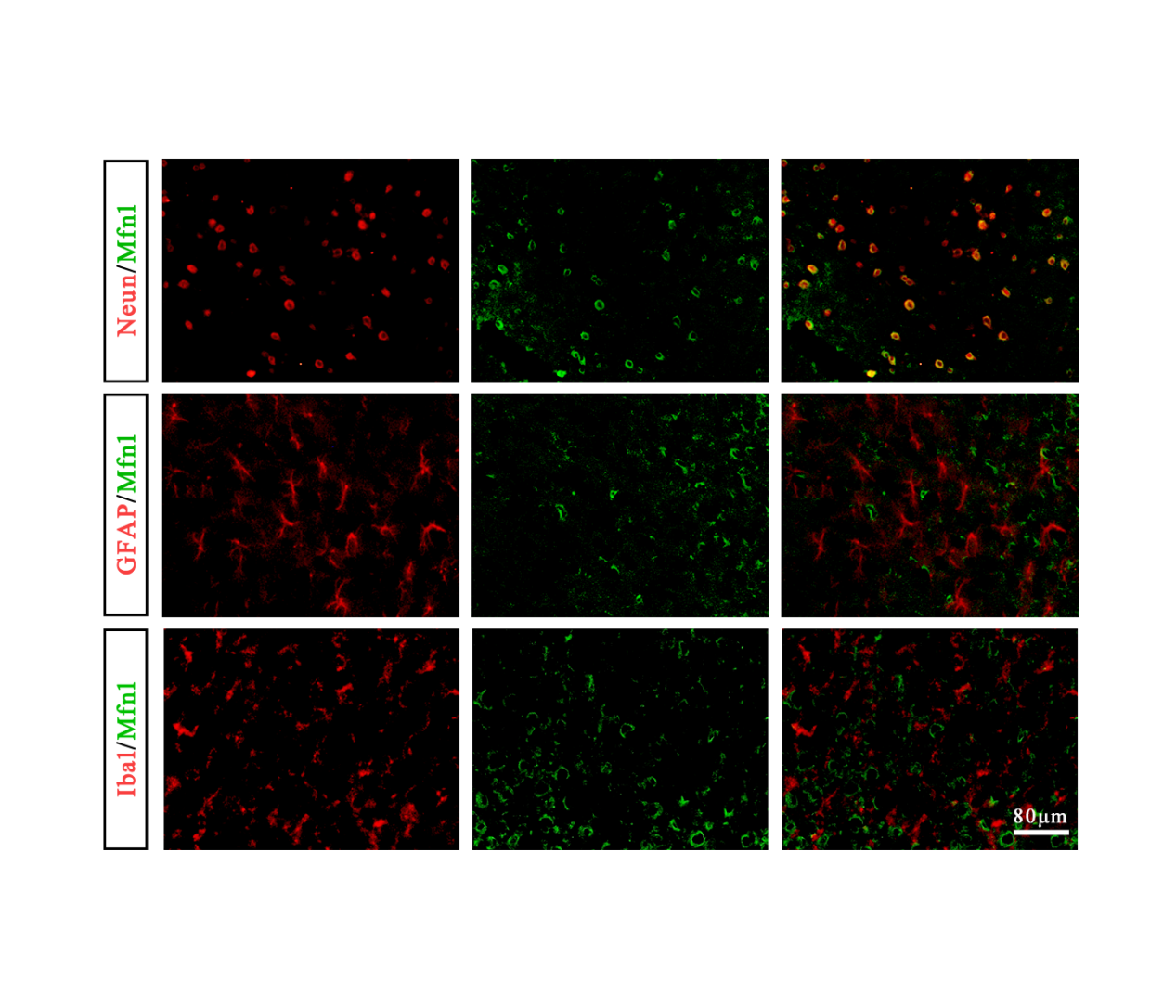


Figure S6. Colocalization of Mfn1 (green) with neurons stained with NeuN (red), microglia stained with Iba-1 (red), astrocytes stained with GFAP (red). Scale bar: 80 µm.


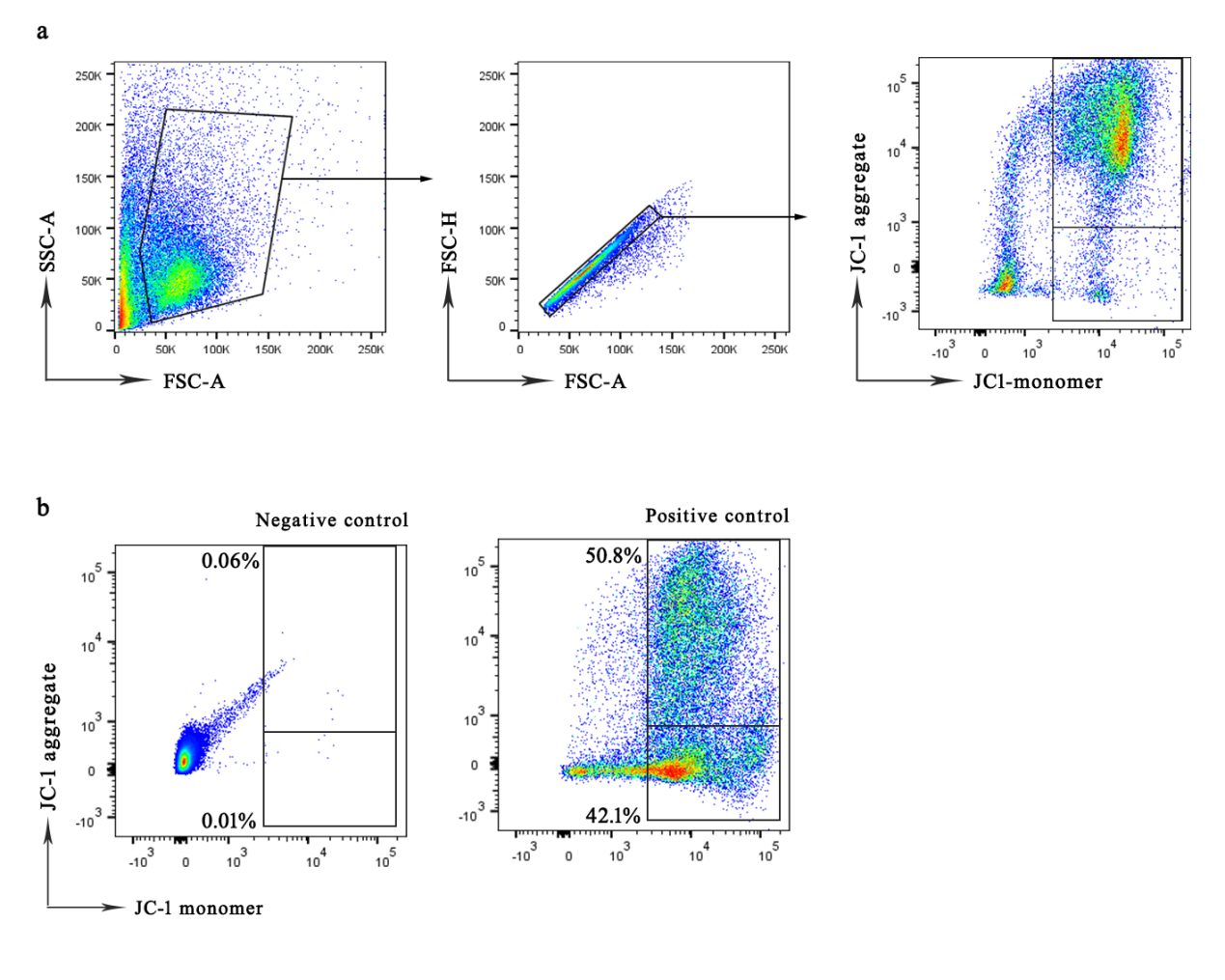


Figure S7. (**a**) Flow cytometry gating strategy of JC-1 fluorescent probes. (**b**) Negative and positive controls for JC-1 staining


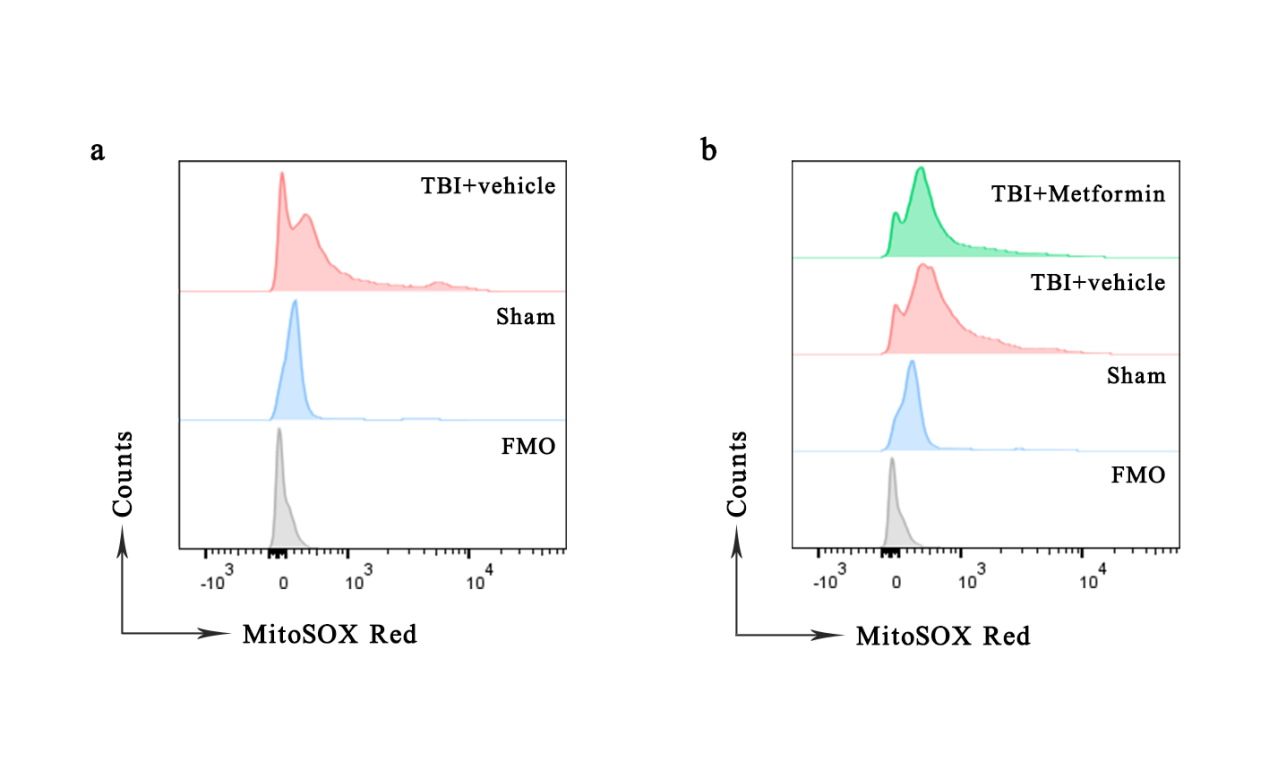


Figure S8. Representative flow cytometry plots show the expression of MitoSOX Red in the ipsilateral hemisphere, corresponding to the data presented in Figure 2h (Panel a) and Figure 4h (Panel b)


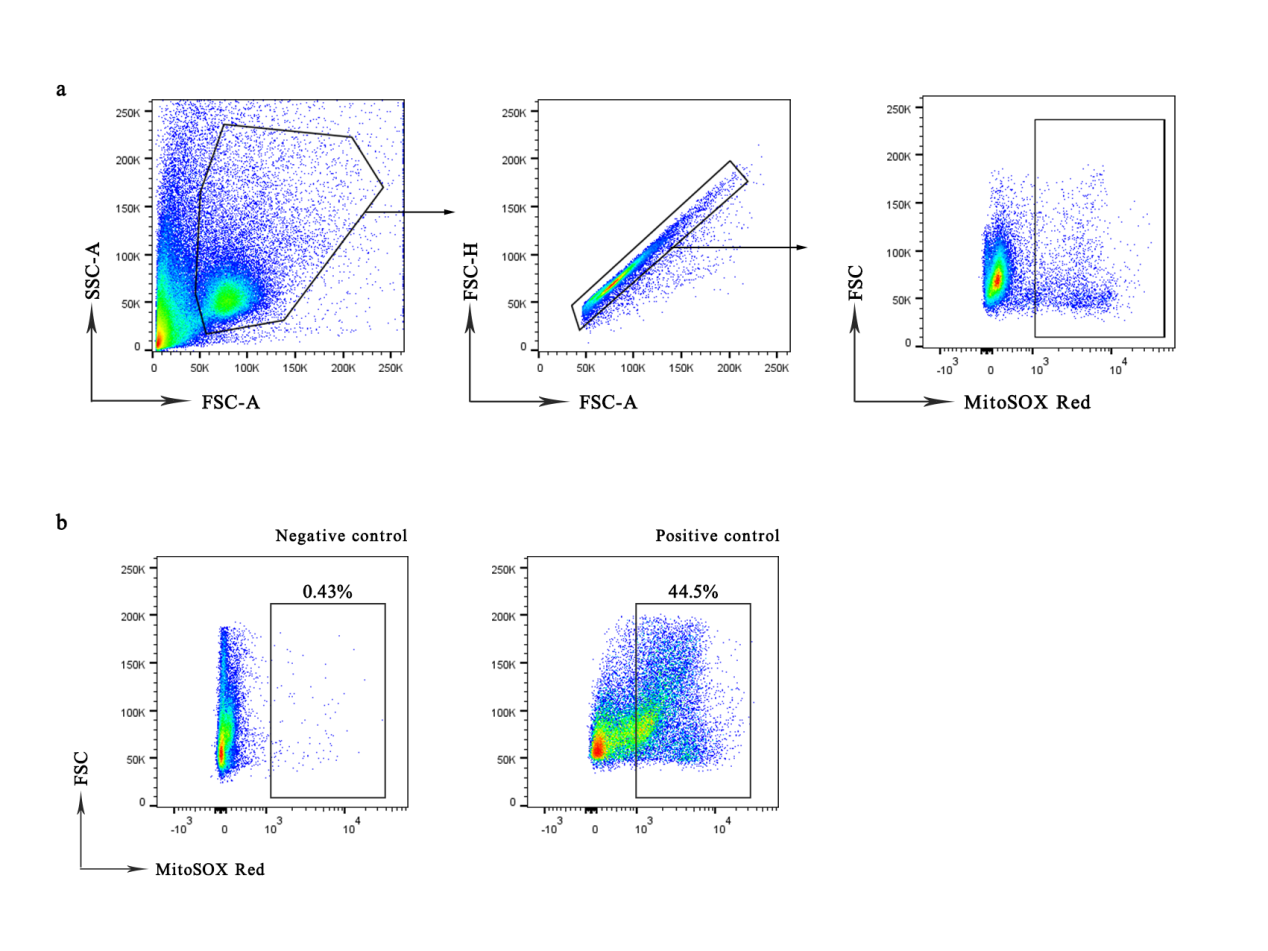


Figure S9. (**a**) Flow cytometry gating strategy of MitoSOX Red. (**b**) Negative and positive controls for MitoSOX staining


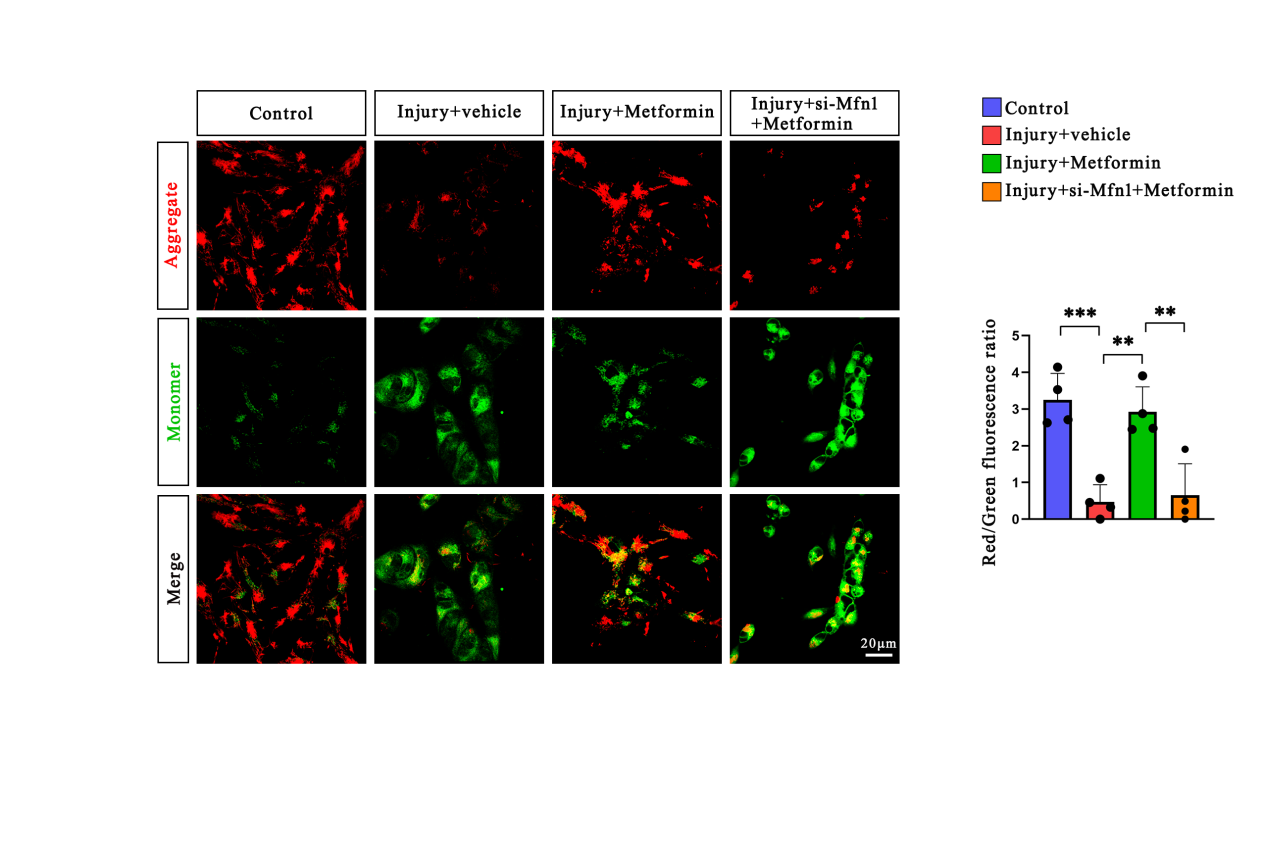


Figure S10. Change in primary neurons’ MMP was analysed by JC-1 staining via confocal laser-scanning microscopy (n=6). Scale bar: 20 µm


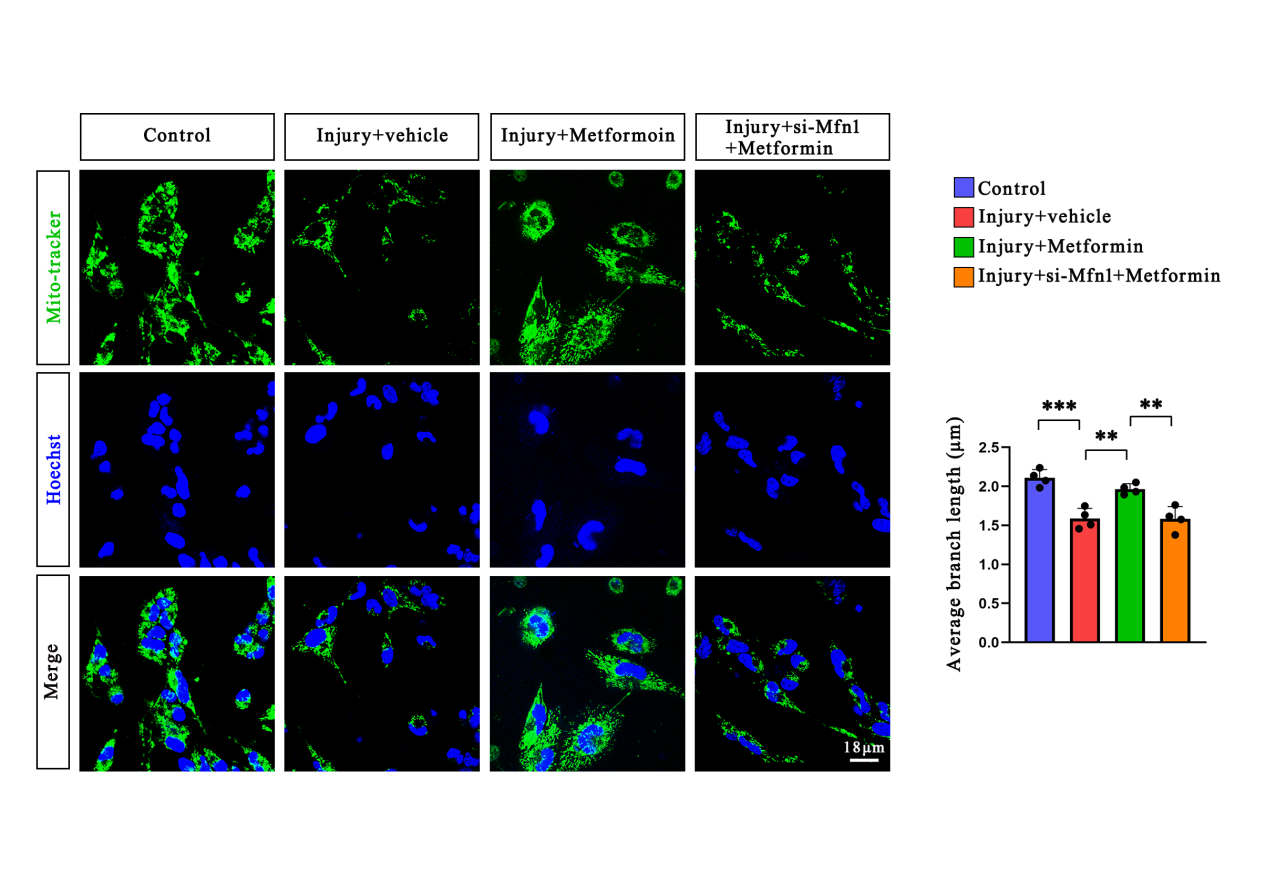


Figure S11. Mitochondria in primary neurons were labeled with MitoTracker Green staining, and mitochondrial morphology was analyzed using confocal laser-scanning microscopy. Scale bar: 18µm


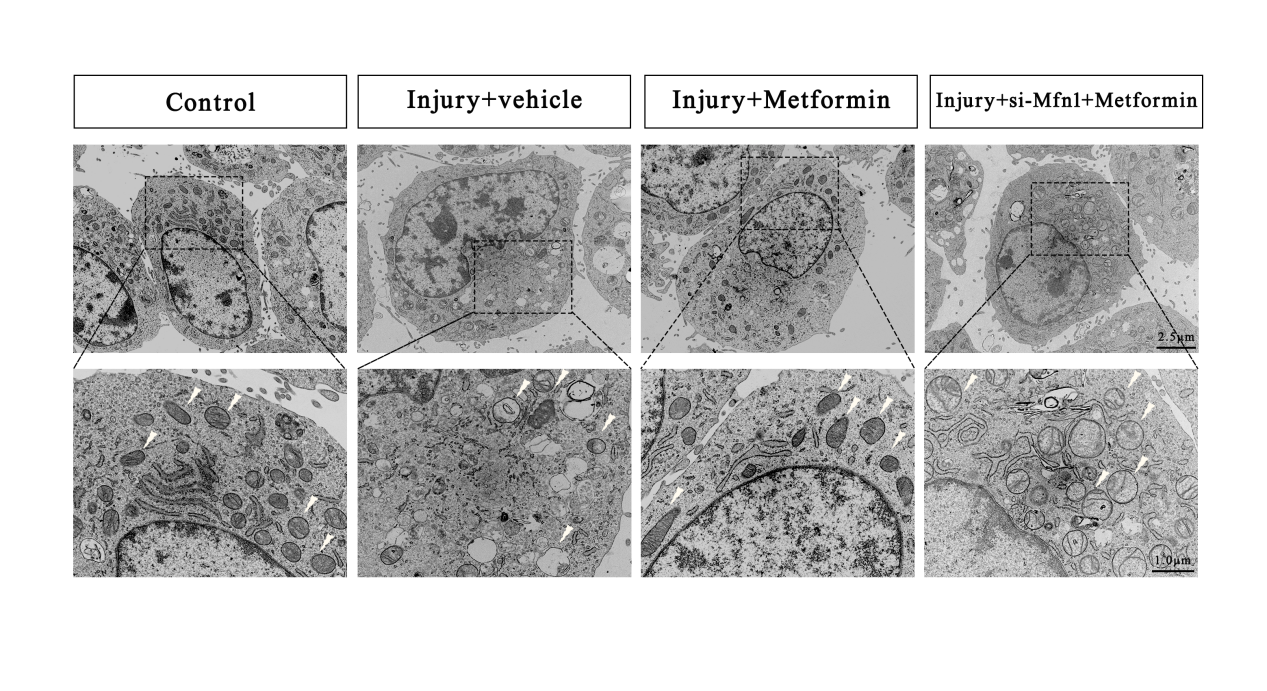


Figure S12. Representative transmission electron microscopy images of primary neurons from indicated groups. The white triangular arrow indicates typical mitochondrial morphology in different groups. Scale bar: 2.5 (top), 1.0 µm (bottom)


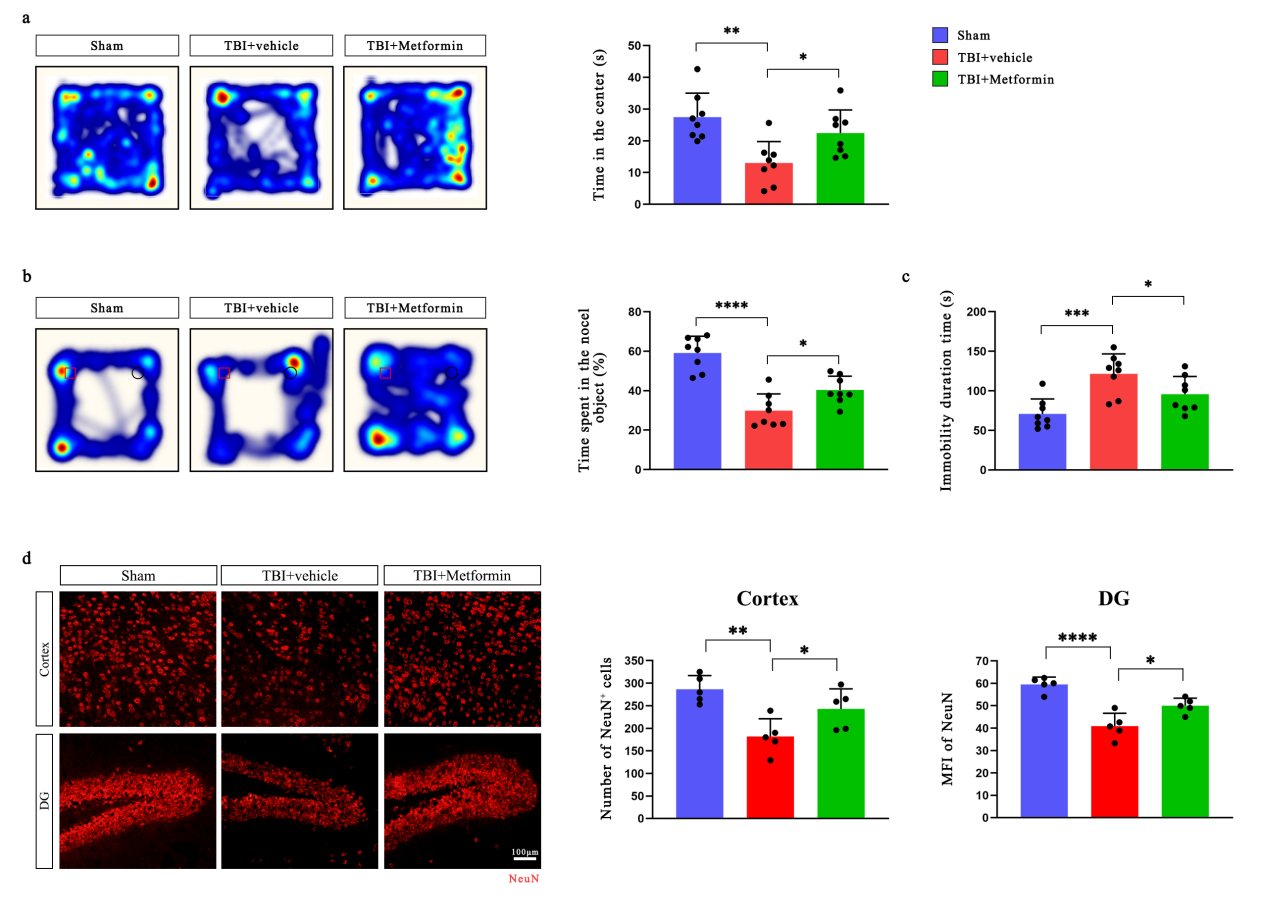


Figure S13. (**a**) Representative thermographic data from the tracking session in the OFT and quantitative analysis of time spent in the center (n=8). (**b**) Representative thermal imaging of mice tracking following NOR test. The discrimination index was analyzed to evaluate memory ability of TBI mice (n=8). (**c**) Immobility time during the TST for the three experimental groups (n=8). (**d**) Representative images of NeuN immunostaining in the ipsilateral hemisphere and quantitative analysis of NeuN mean fluorescence intensity (MFI) in the ipsilateral dentate gyrus (DG) and NeuN^+^ neurons in the cortex regions 1 month following TBI (n=4-6). Scale bar: 100 µm. **p* < 0.05, ***p* < 0.01, ****p* < 0.001, *****p* < 0.0001


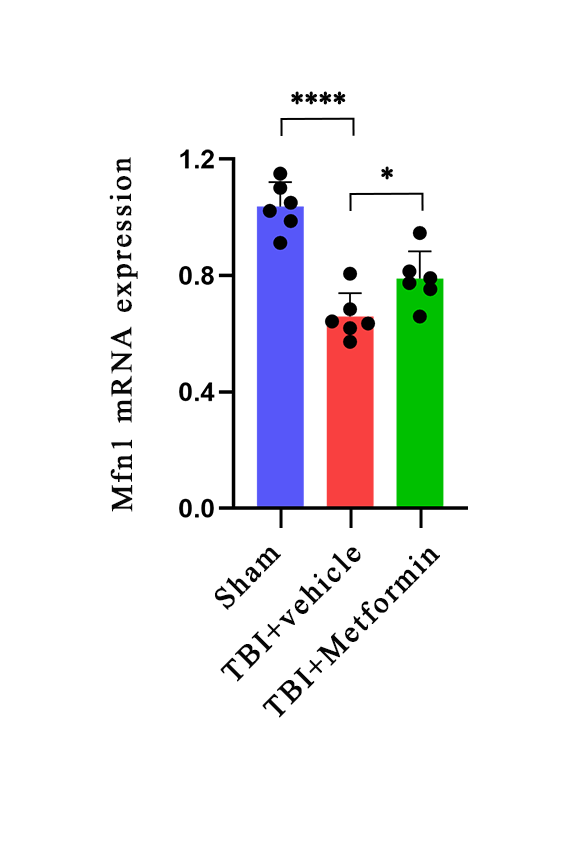


Figure S14. Real-time PCR analysis of Mfn1 mRNA expression in the cortex of TBI mice following metformin treatment (n=6). **p* < 0.05, *****p* < 0.0001


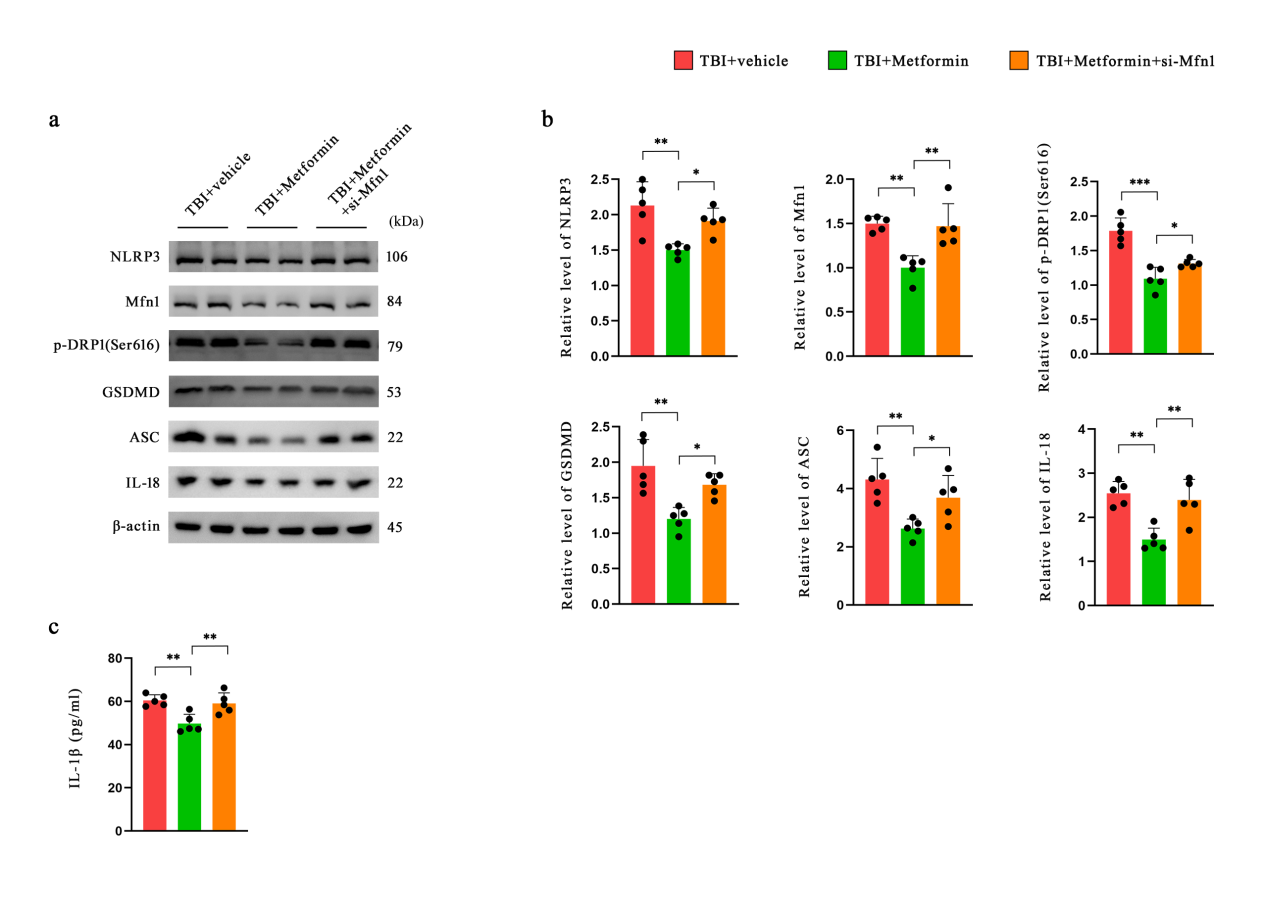


Figure S15. (**a, b**) Protein levels of NLRP3 inflammasome biomarkers (NLRP3, ASC, IL-18), GSDMD and mitochondrial dynamics biomarkers (Mfn1, p-Drp1 (Ser616)) in the primary neurons after scratch assay (n=5). (**c**) Elisa analyses of IL-1β in the primary neurons after scratch assay from indicated groups (n=5). **p* < 0.05, ***p* < 0.01, ****p* < 0.001, *****p* < 0.0001

Fig S16. Protein levels of Mfn1 in the cortex of mice from indicated groups (n=4). ****p* < 0.001
