# Supplementary figures and images for: Targeting mitofusin 1-mediated mitochondrial dynamics to suppress neuroinflammation and pyroptosis after traumatic brain injury
Source: Burns Trauma. 2026 Jan 28;14:tkag011. doi: 10.1093/burnst/tkag011 (PMC13152094; doi:10.1093/burnst/tkag011)

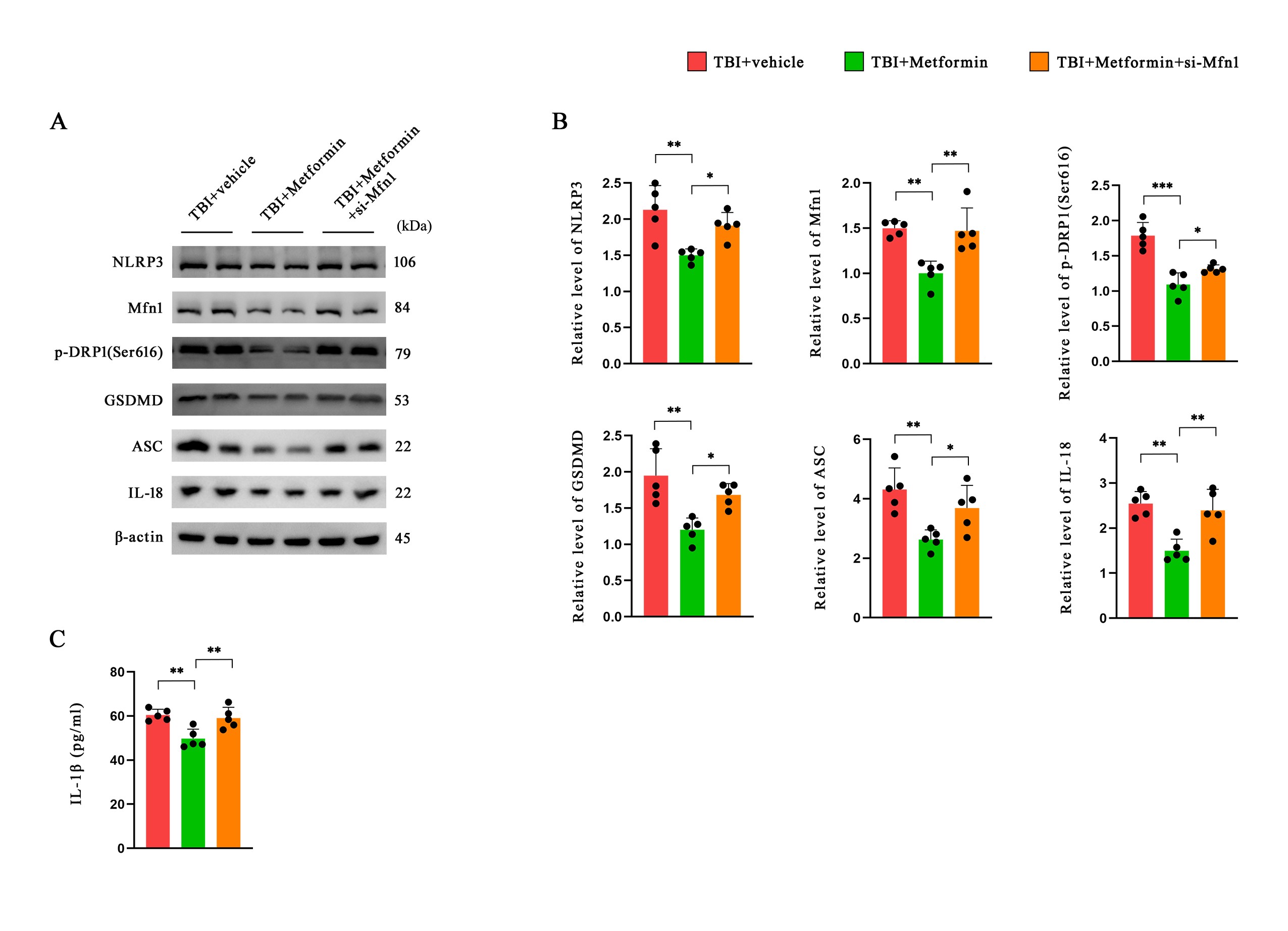

Supplement: tkag011_Supplemental_Files [file tkag011_supplemental_files.zip › Figure_S10_tkag011.jpg]

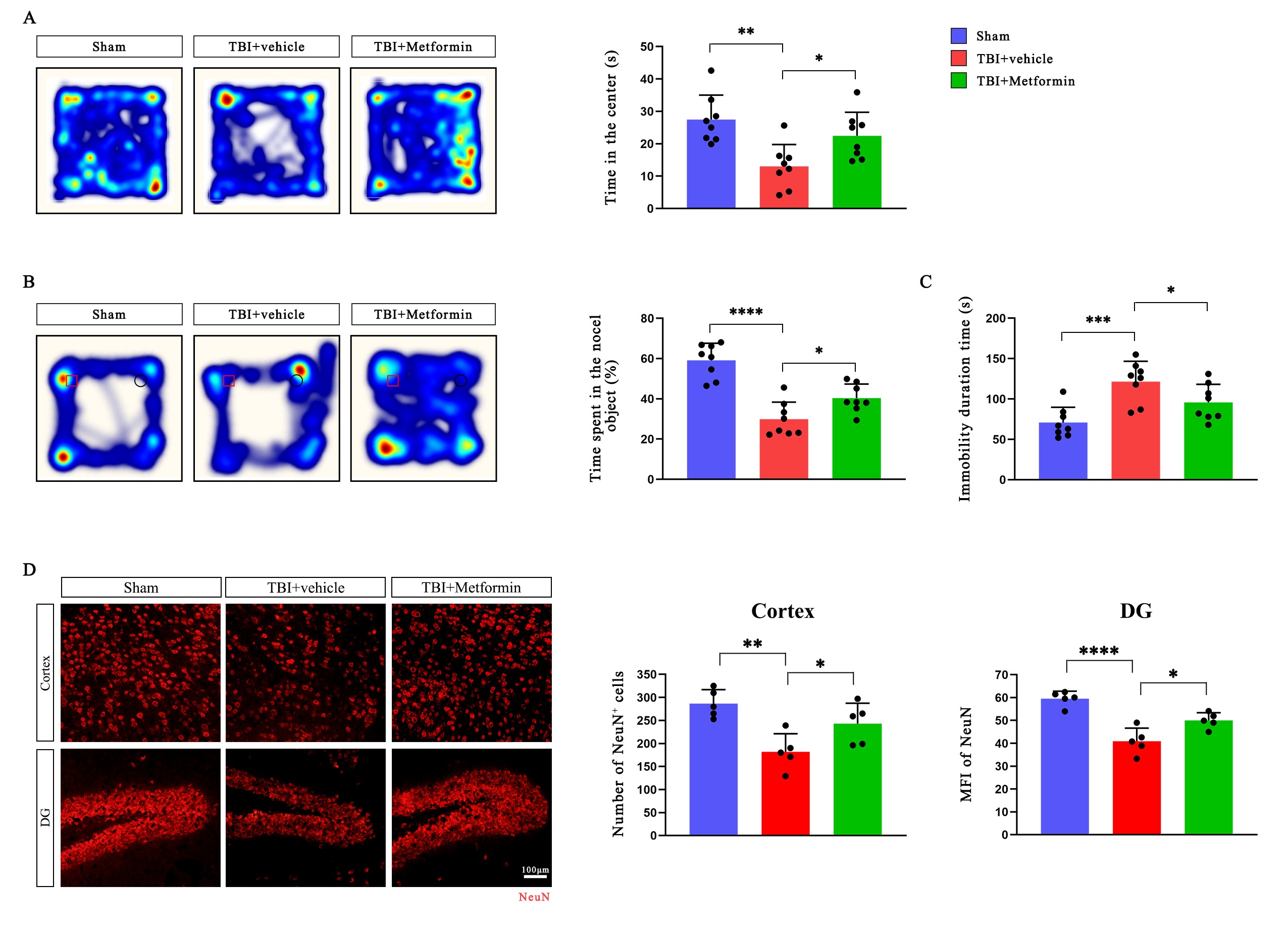

Supplement: tkag011_Supplemental_Files [file tkag011_supplemental_files.zip › Figure_S11_tkag011.jpg]

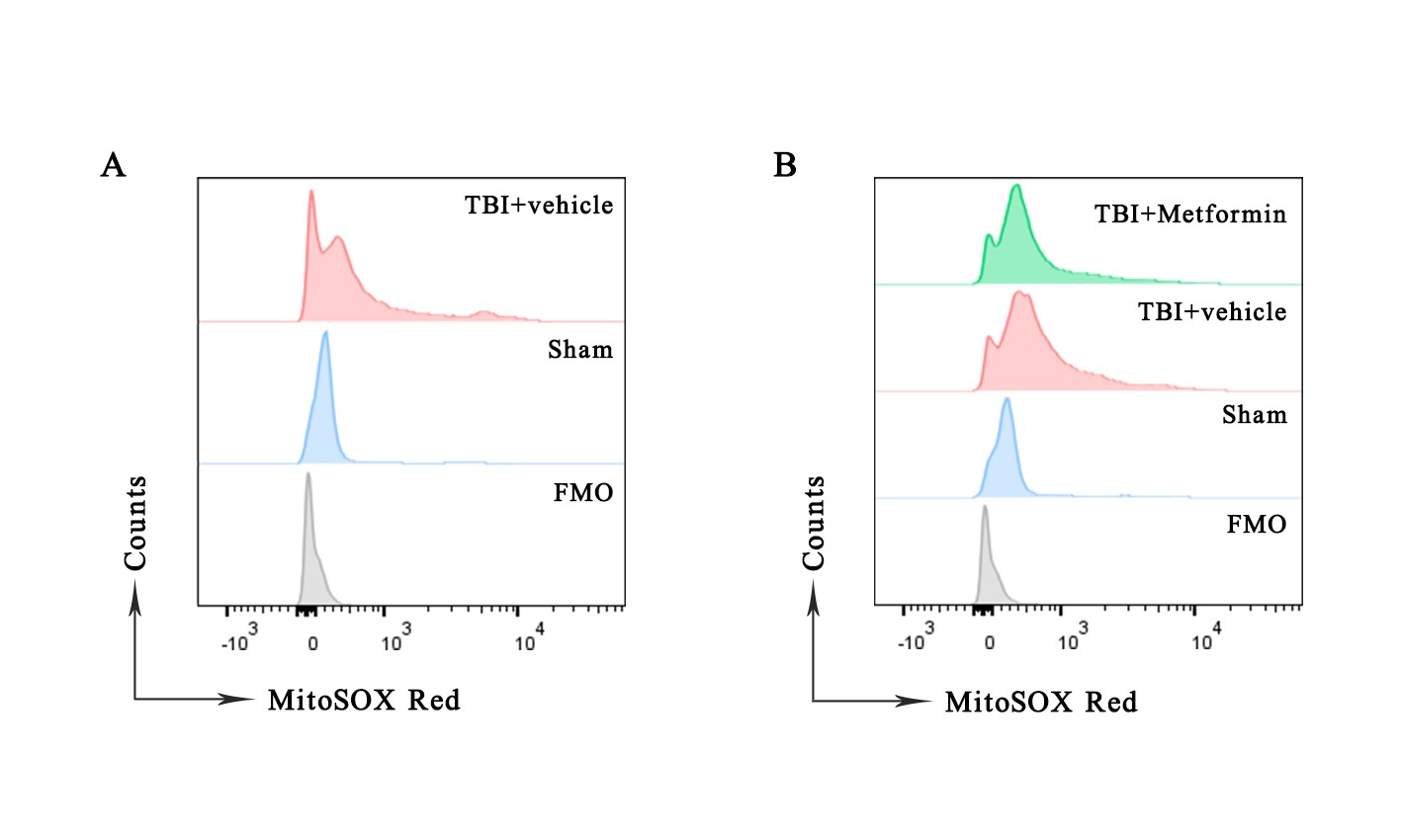

Supplement: tkag011_Supplemental_Files [file tkag011_supplemental_files.zip › Figure_S12_tkag011.jpg]

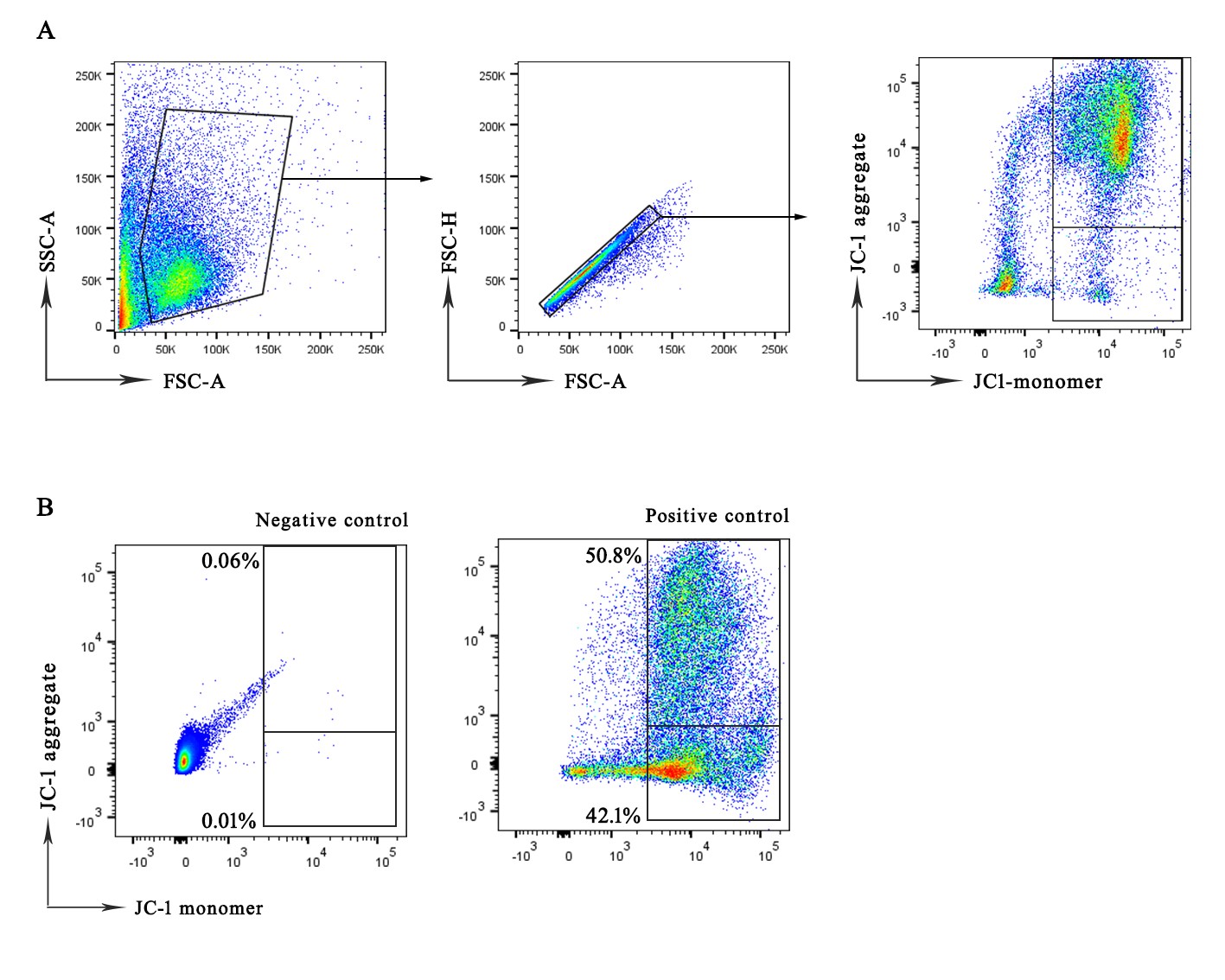

Supplement: tkag011_Supplemental_Files [file tkag011_supplemental_files.zip › Figure_S13_tkag011.jpg]

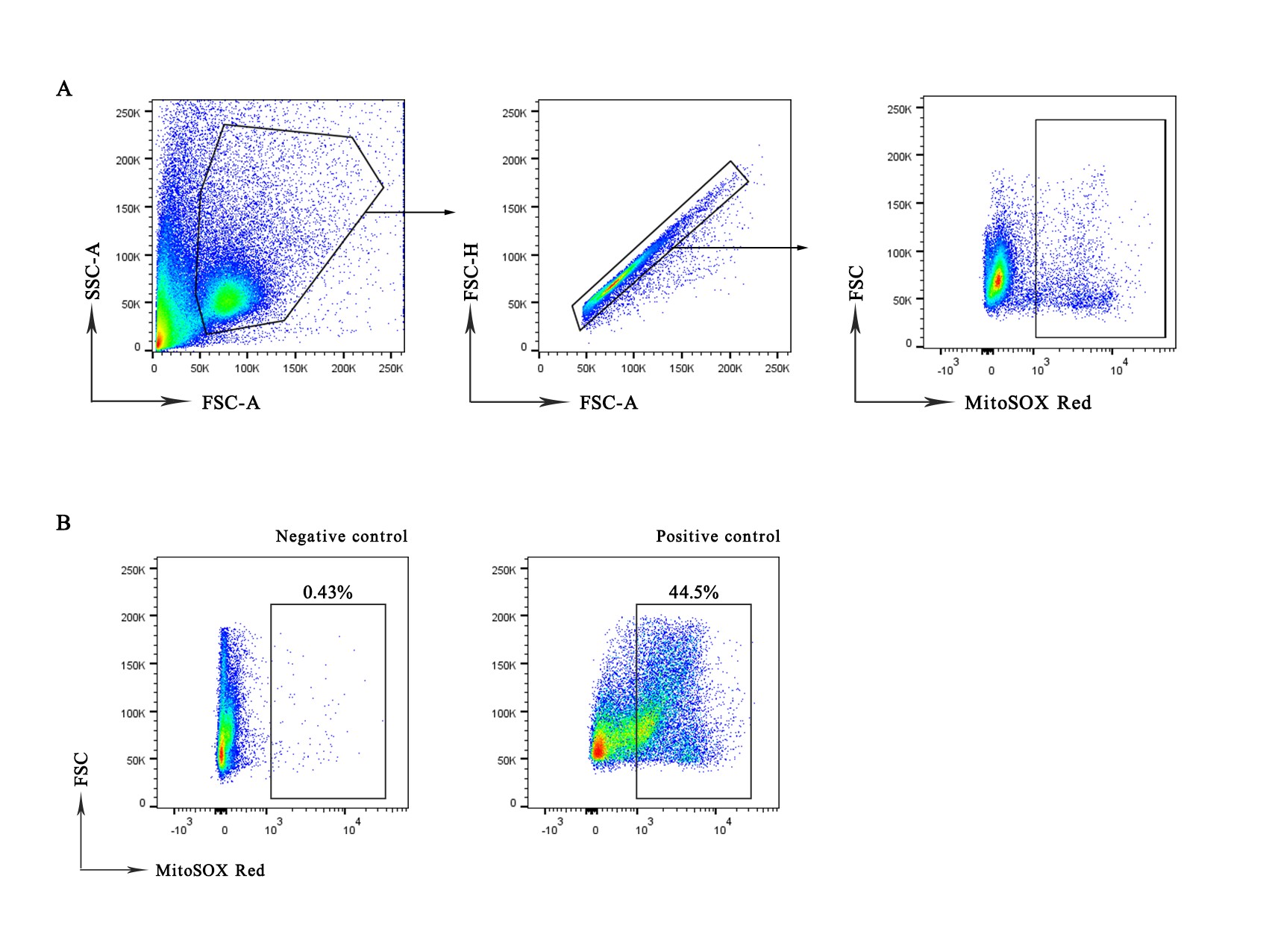

Supplement: tkag011_Supplemental_Files [file tkag011_supplemental_files.zip › Figure_S14_tkag011.jpg]

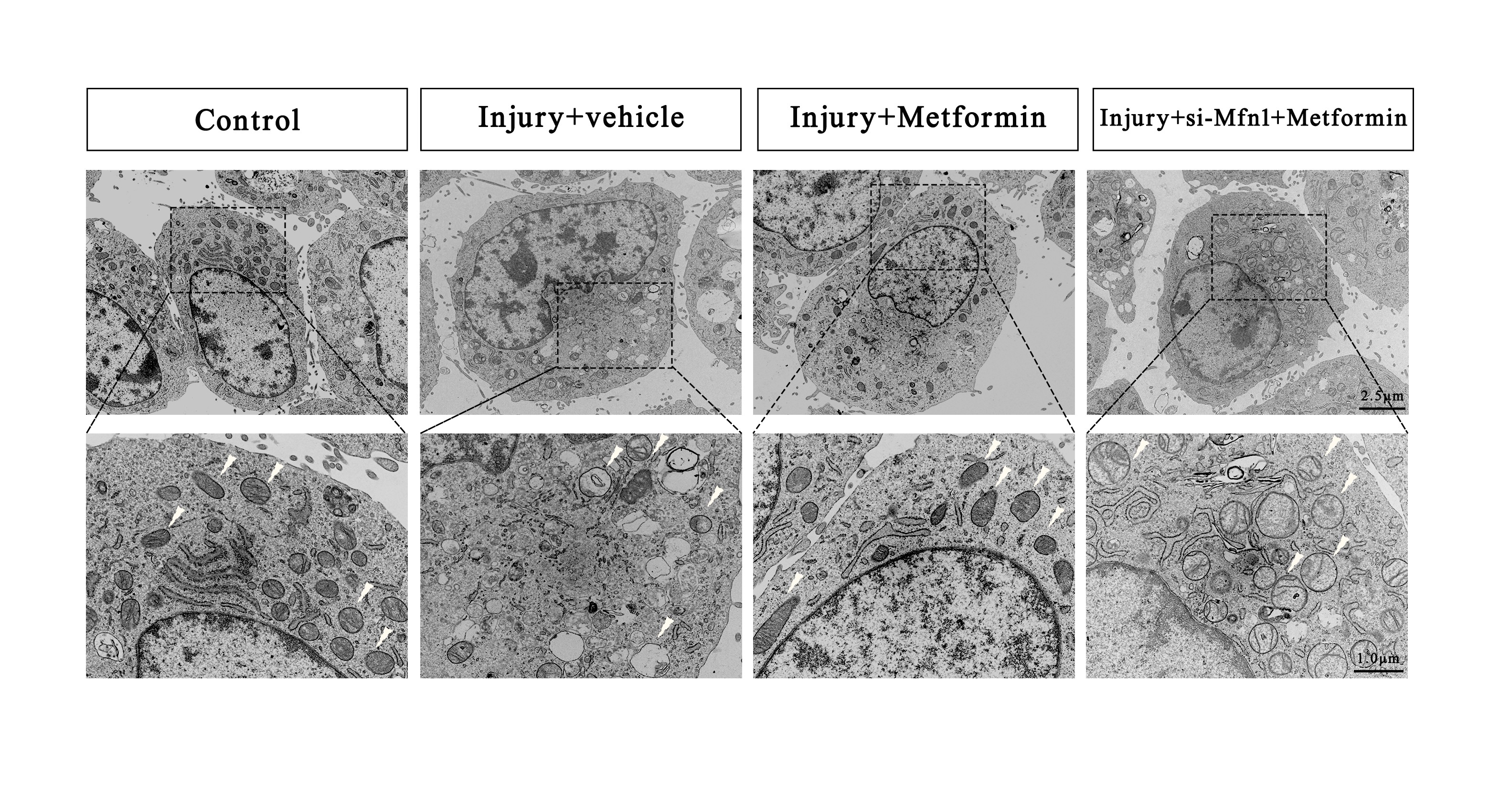

Supplement: tkag011_Supplemental_Files [file tkag011_supplemental_files.zip › Figure_S15_tkag011.jpg]

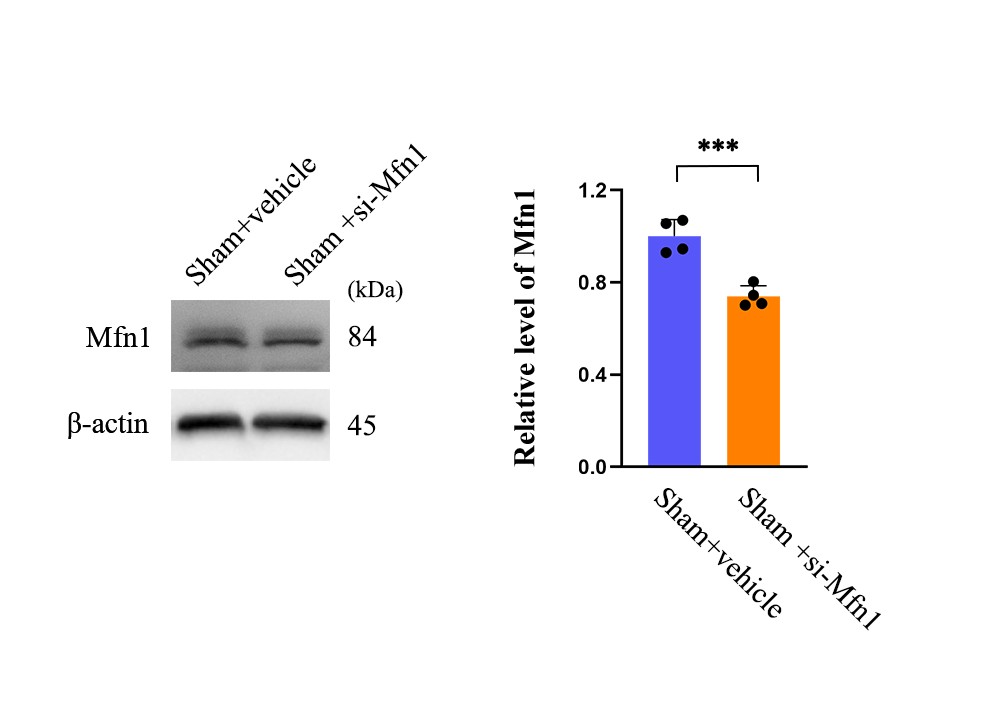

Supplement: tkag011_Supplemental_Files [file tkag011_supplemental_files.zip › Figure_S16_tkag011.jpg]

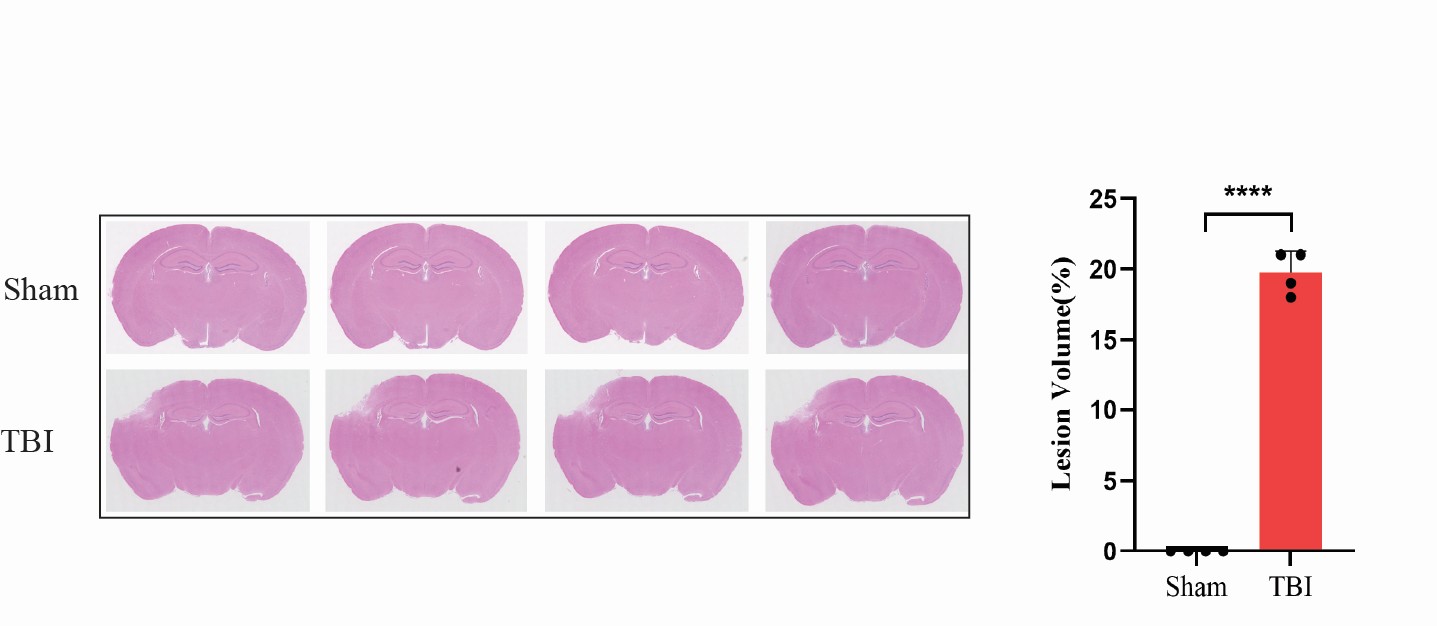

Supplement: tkag011_Supplemental_Files [file tkag011_supplemental_files.zip › Figure_S1_tkag011.jpg]

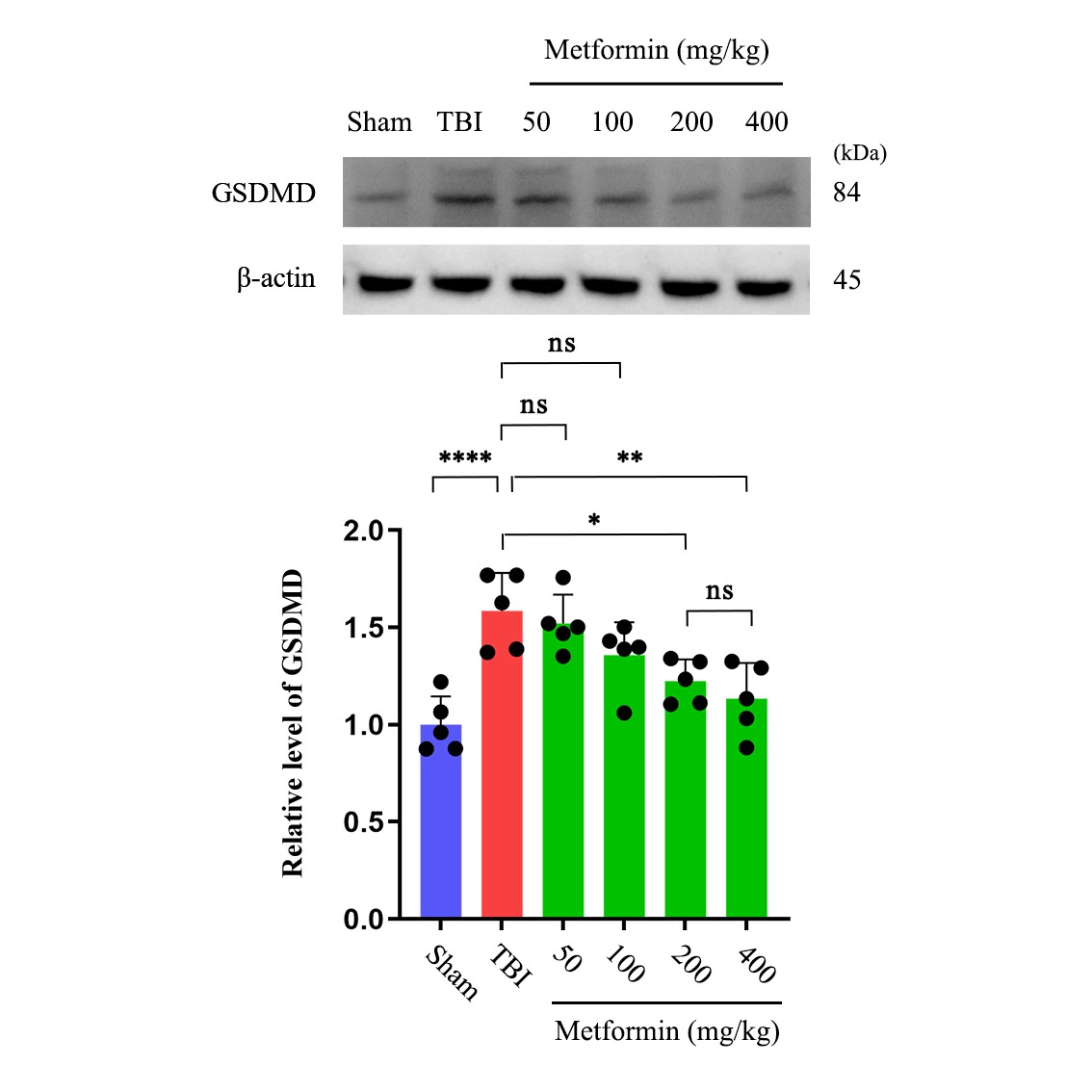

Supplement: tkag011_Supplemental_Files [file tkag011_supplemental_files.zip › Figure_S2_tkag011.jpg]

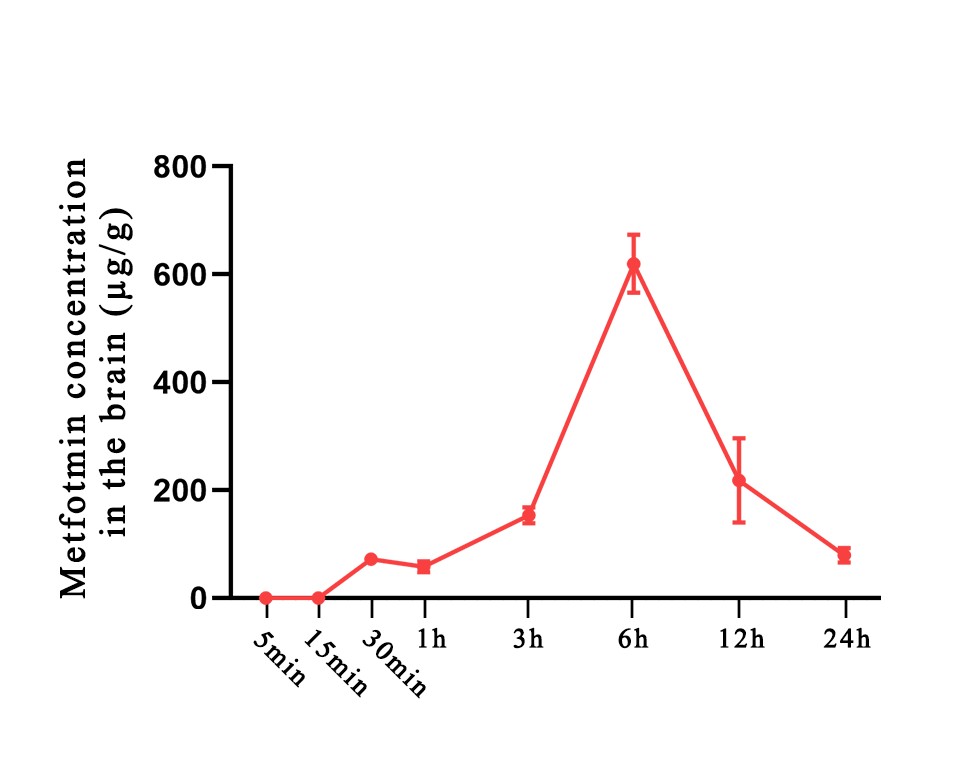

Supplement: tkag011_Supplemental_Files [file tkag011_supplemental_files.zip › Figure_S3_tkag011.jpg]

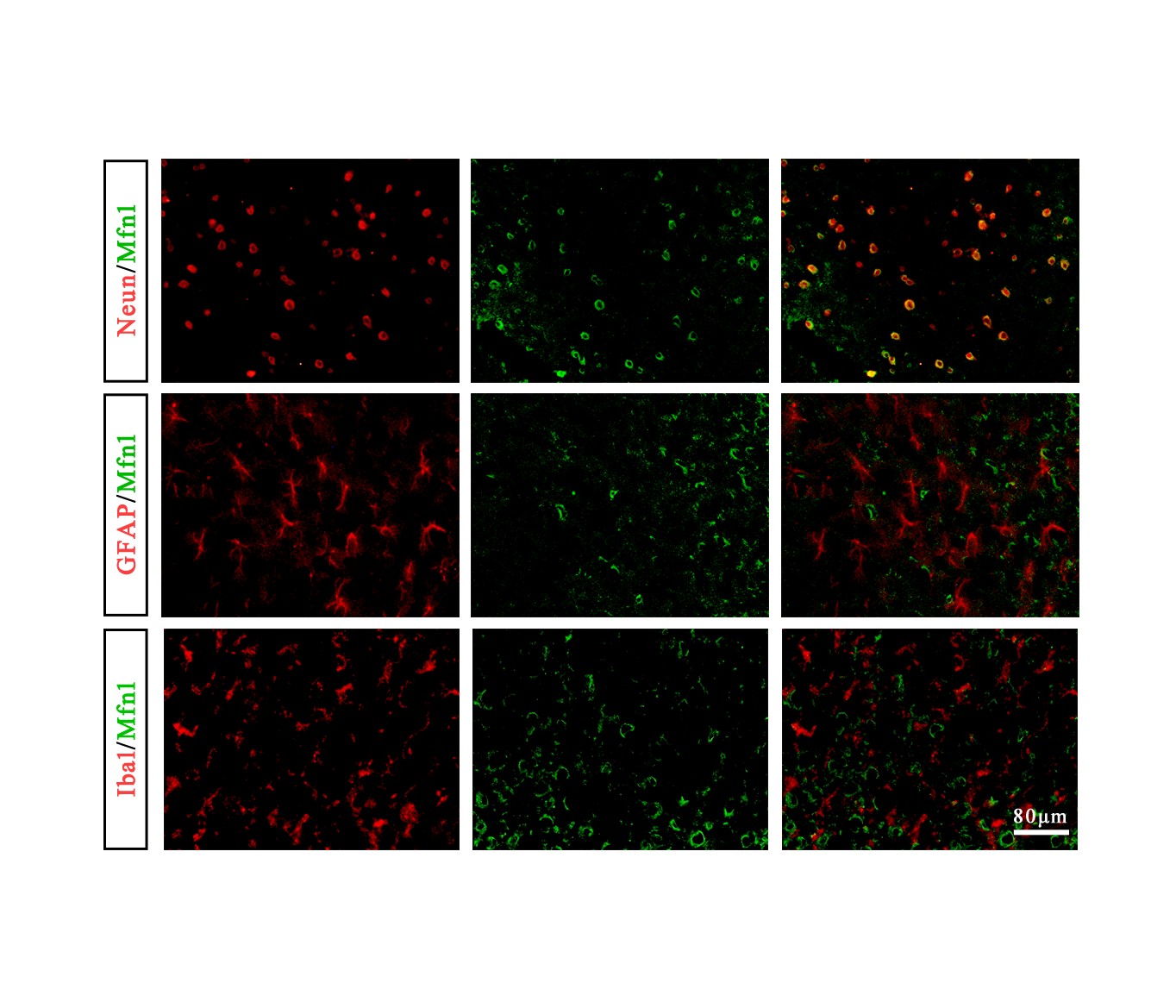

Supplement: tkag011_Supplemental_Files [file tkag011_supplemental_files.zip › Figure_S4_tkag011.jpg]

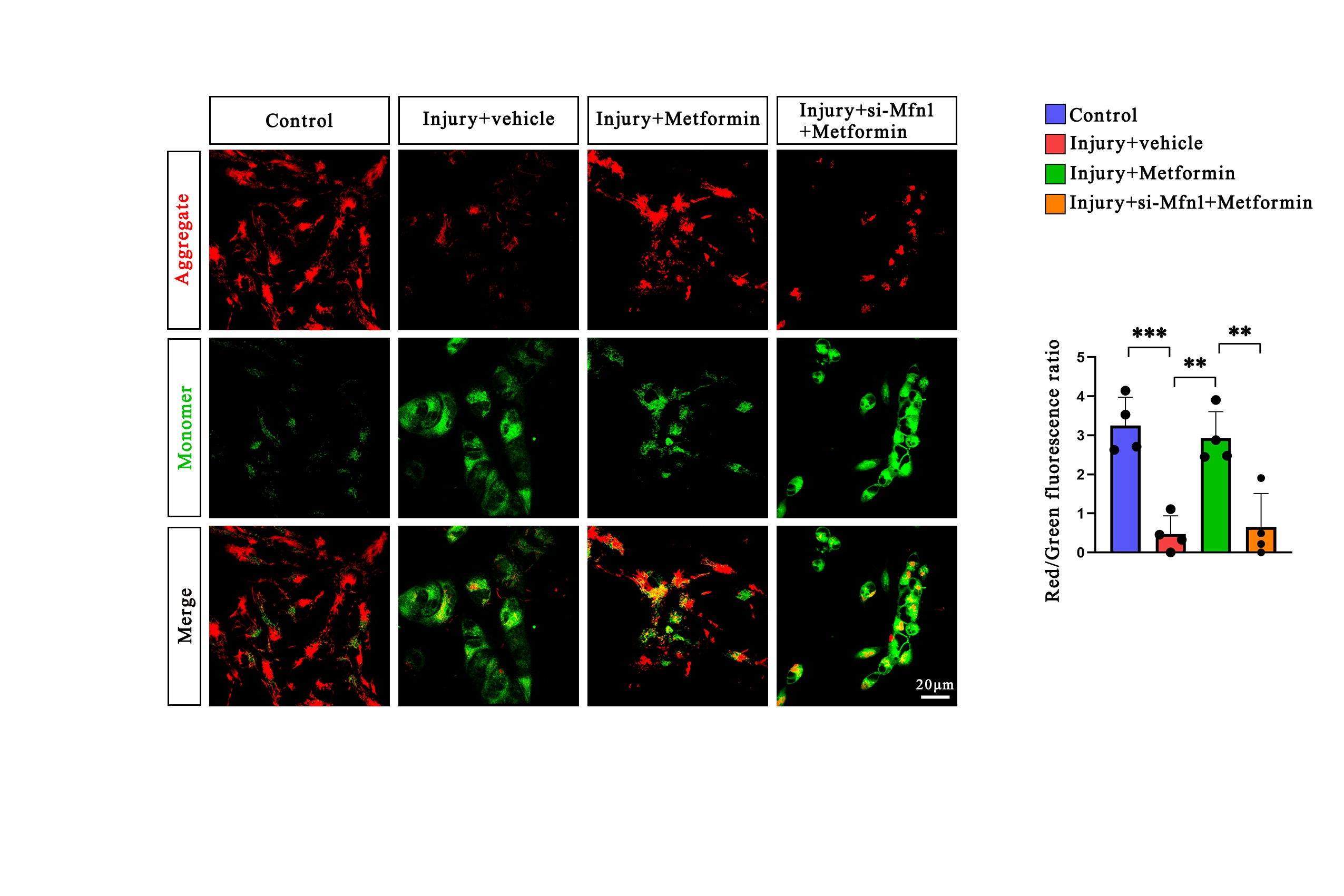

Supplement: tkag011_Supplemental_Files [file tkag011_supplemental_files.zip › Figure_S5_tkag011.jpg]

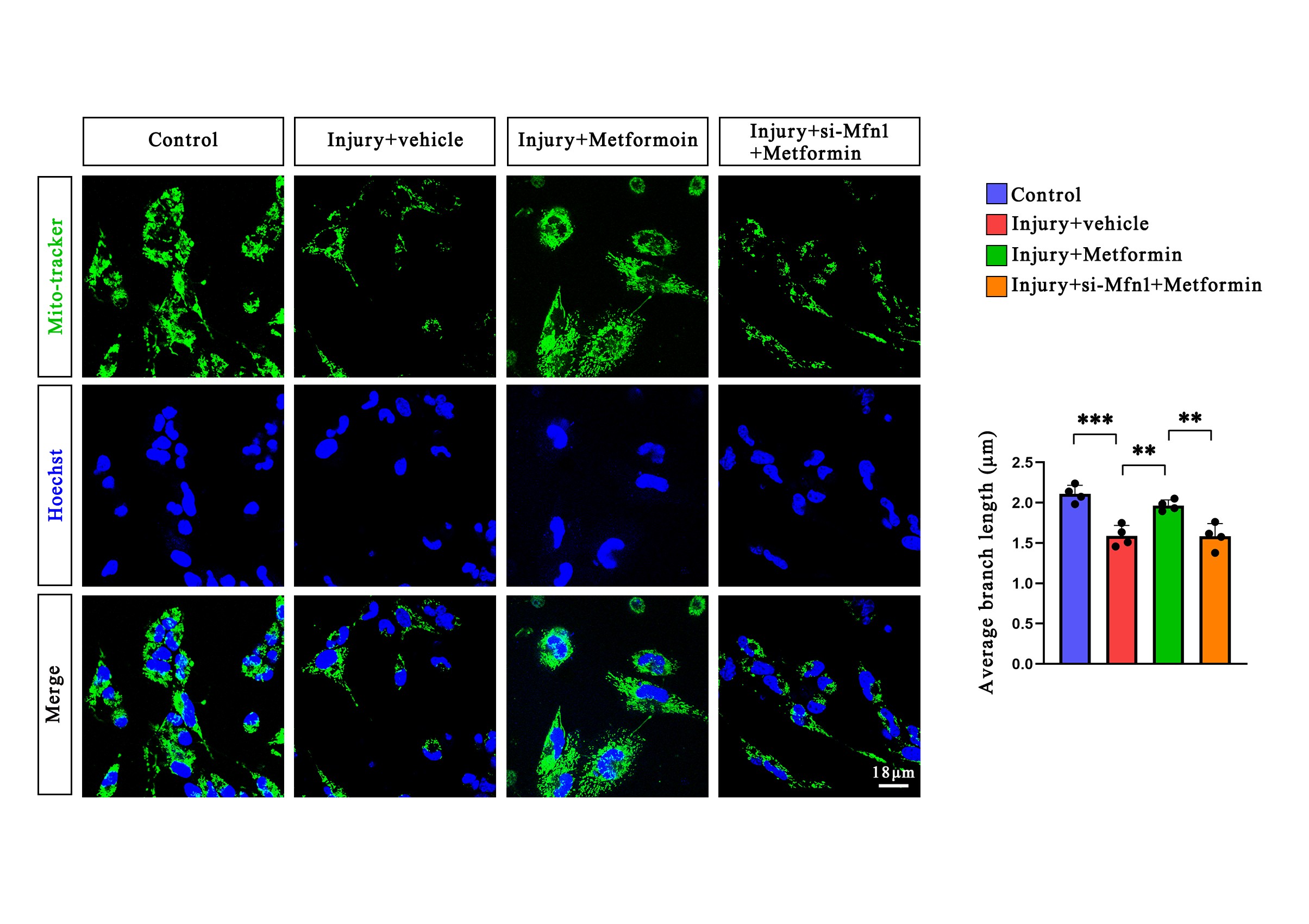

Supplement: tkag011_Supplemental_Files [file tkag011_supplemental_files.zip › Figure_S6_tkag011.jpg]

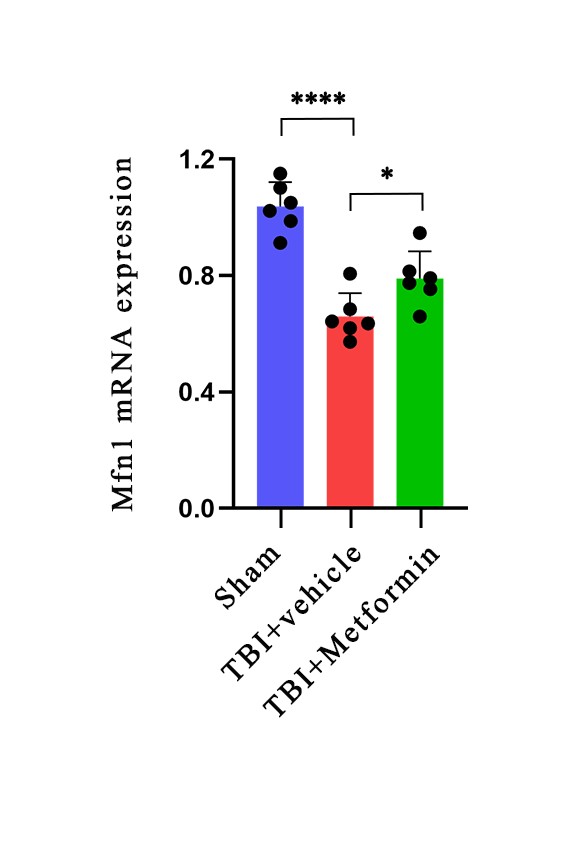

Supplement: tkag011_Supplemental_Files [file tkag011_supplemental_files.zip › Figure_S7_tkag011.jpg]

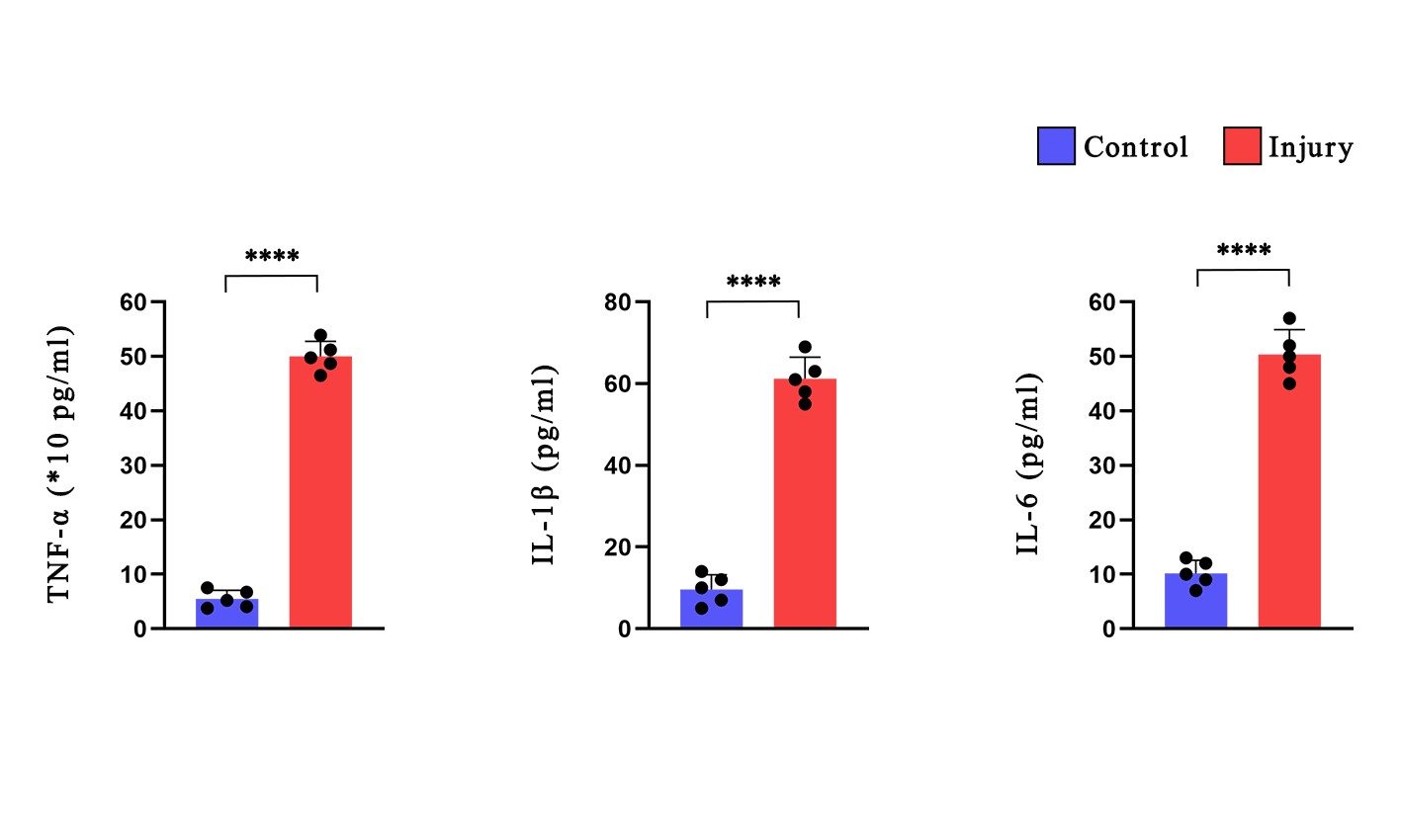

Supplement: tkag011_Supplemental_Files [file tkag011_supplemental_files.zip › Figure_S8_tkag011.jpg]

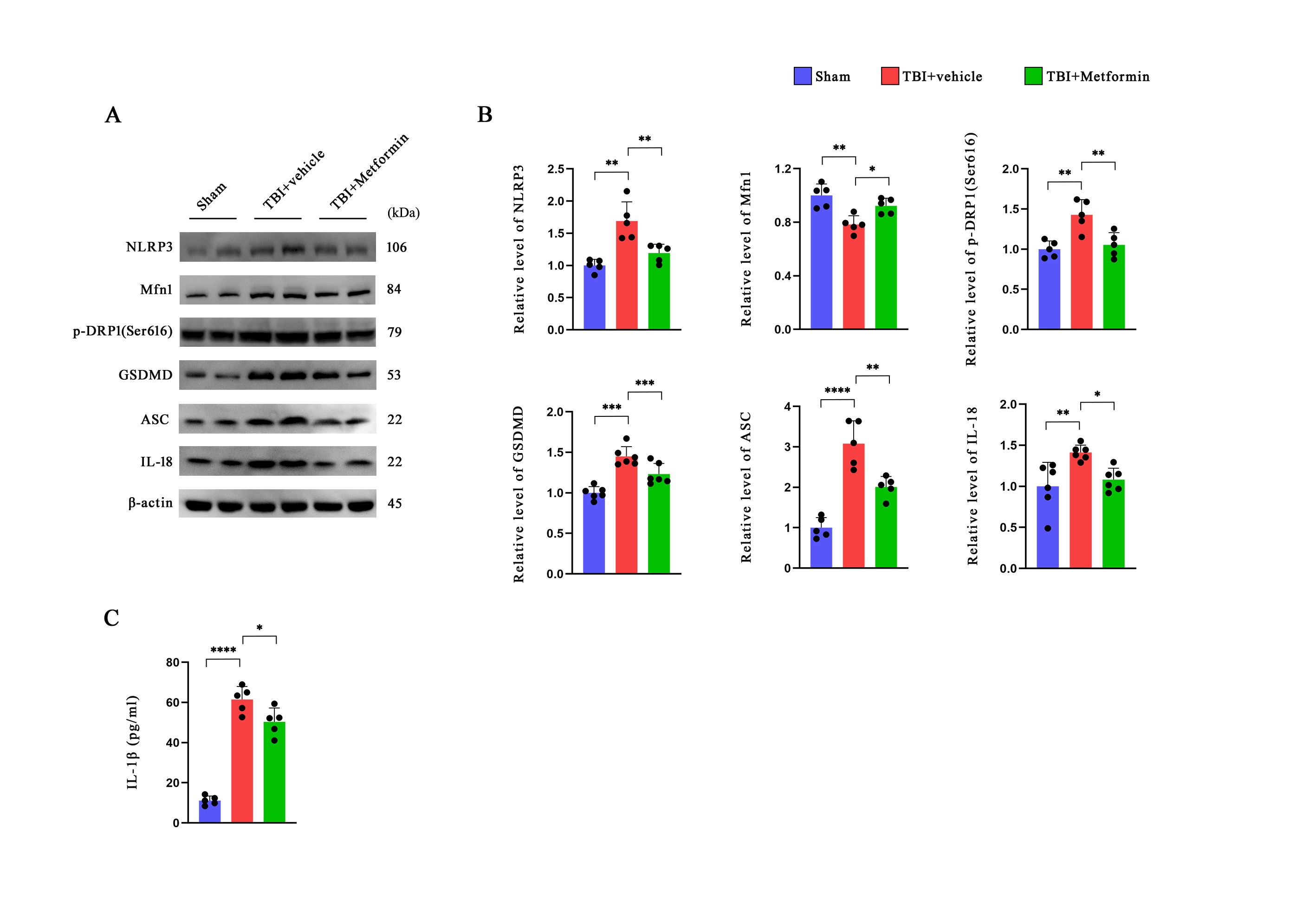

Supplement: tkag011_Supplemental_Files [file tkag011_supplemental_files.zip › Figure_S9_tkag011.jpg]
