# Supplementary material for: Targeting mitofusin 1-mediated mitochondrial dynamics to suppress neuroinflammation and pyroptosis after traumatic brain injury
Source: Burns Trauma. 2026 Jan 28;14:tkag011. doi: 10.1093/burnst/tkag011 (PMC13152094; doi:10.1093/burnst/tkag011)
Supplement: tkag011_Supplemental_Files [file tkag011_supplemental_files.zip › 1.1Supplementary_figure_legends_tkag011.doc]

**Supplementary figure legends**

Figure S1 Coronal HE-stained sections from mice in the sham group 12 hours after CCI impact. The bar chart shows severe tissue defects in the cerebral cortex of mice 12 hours after CCI, and the percentage of the brain defect area varied little among the different sham mice, demonstrating the stability of the CCI model (n=4). ****p < 0.0001.

Figure S2 Protein levels of GSDMD in the ipsilateral cortex of mice in the sham group and TBI group treated with metformin at doses of 50, 100, 200 and 400 mg/kg (n=5). *p < 0.05, **p < 0.01, ****p < 0.0001.

Figure S3 Metformin concentration in the brain tissue of the TBI mice within 24 hours (n=3).

Figure S4 Colocalization of Mfn1 (green) with neurons stained with NeuN (red), microglia stained with Iba-1 (red), and astrocytes stained with GFAP (red). Scale bar=80 µm.

Figure S5 Changes in the MMP of primary neurons were analyzed by JC-1 staining via confocal laser-scanning microscopy (n=6). Scale bar = 20 µm.

Figure S6 Mitochondria in primary neurons were labeled with MitoTracker Green, and mitochondrial morphology was analyzed using confocal laser-scanning microscopy. Scale bar = 18 µm.

Figure S7 Real-time PCR analysis of Mfn1 mRNA expression in the cortex of TBI mice following metformin treatment (n=6). *p < 0.05, ****p < 0.0001.

Figure S8 ELISA analyses of proinflammatory cytokines (e.g., TNF-α, IL-1β and IL-6) in primary neurons from the indicated groups (n=5). ****p < 0.0001.

Figure S9 A, B Protein levels of NLRP3 inflammasome biomarkers (e.g., NLRP3, ASC, and IL-18), GSDMD and mitochondrial dynamics biomarkers (e.g., Mfn1 and p-Drp1 (Ser616)) in primary neurons after the scratch assay (n=5). C ELISA analyses of IL-1β in primary neurons from the indicated groups after the scratch assay (n=5). *p < 0.05, **p < 0.01, ***p < 0.001, ****p < 0.0001.

Figure S10 A, B Protein levels of NLRP3 inflammasome biomarkers (e.g., NLRP3, ASC and IL-18), GSDMD and mitochondrial dynamics biomarkers (e.g., Mfn1 and p-Drp1 (Ser616)) in primary neurons after the scratch assay (n=5). C ELISA analyses of IL-1β in primary neurons from the indicated groups after the scratch assay (n=5). *p < 0.05, **p < 0.01, ***p < 0.001, ****p < 0.0001.

Figure S11 A Representative thermographic data from the tracking session in the OF test and quantitative analysis of time spent in the center (n=8). B Representative thermal images of mice following the novel object recognition test. The discrimination index was analyzed to evaluate the memory ability of the TBI mice (n =8). C Immobility time during the tail suspension test for the three experimental groups (n=8). D Representative images of NeuN immunostaining in the ipsilateral hemisphere and quantitative analysis of the mean fluorescence intensity (MFI) of NeuN in the ipsilateral dentate gyrus (DG) and NeuN+ neurons in the cortex 1 month after TBI (n=4–6). Scale bar = 100 µm. *p < 0.05, **p < 0.01, ***p < 0.001, ****p < 0.0001.

Figure S12 Representative flow cytometry plots showing the expression of MitoSOX Red in the ipsilateral hemisphere, corresponding to the data presented in Figure 2H (Panel A) and Figure 4H (Panel B).

Figure S13 A Flow cytometry gating strategy for JC-1 fluorescent probes. B Negative and positive controls for JC-1 staining.

Figure S14 A Flow cytometry gating strategy for MitoSOX Red. B Negative and positive controls for MitoSOX staining.

Figure S15 Representative transmission electron microscopy images of primary neurons from the indicated groups. The white triangular arrow indicates typical mitochondrial morphology in the different groups. Scale bars = 2.5 (top) and 1.0 µm (bottom).

Figure S16 Protein levels of Mfn1 in the cortex of mice from the indicated groups (n=4). ***p < 0.001.
